# Sustained In Vitro and In Vivo Delivery of Metformin from Plant Pollen-Derived Composite Microcapsules

**DOI:** 10.3390/pharmaceutics13071048

**Published:** 2021-07-09

**Authors:** Noha M. Meligi, Amro K. F. Dyab, Vesselin N. Paunov

**Affiliations:** 1 Zoology Department, Faculty of Science, Minia University, Minia 61519, Egypt; noha.melege@mu.edu.eg; 2 Colloids & Advanced Materials Group, Chemistry Department, Faculty of Science, Minia University, Minia 61519, Egypt; amro.dyab@mu.edu.eg; 3 Department of Chemistry, School of Sciences and Humanities, Nazarbayev University, Nursultan 010000, Kazakhstan

**Keywords:** metformin, antidiabetic, plant spores, sporopollenin, alginate, beads, biochemical parameters, histopathology

## Abstract

We developed a dual microencapsulation platform for the type 2 diabetes drug metformin (MTF), which is aimed to increase its bioavailability. We report the use of *Lycopodium clavatum* sporopollenin (LCS), derived from their natural spores, and raw *Phoenix dactylifera* L. (date palm) pollens (DPP) for MTF microencapsulation. MTF was loaded into LCS and DPP via a vacuum and a novel method of hydration-induced swelling. The loading capacity (LC) and encapsulation efficiency (EE) percentages for MTF-loaded LCS and MTF-loaded DPP microcapsules were 14.9% ± 0.7, 29.8 ± 0.8, and 15.2% ± 0.7, 30.3 ± 1.0, respectively. The release of MTF from MTF-loaded LCS microcapsules was additionally controlled by re-encapsulating the loaded microcapsules into calcium alginate (ALG) microbeads via ionotropic gelation, where the release of MTF was found to be significantly slower and pH-dependent. The pharmacokinetic parameters, obtained from the in vivo study, revealed that the relative bioavailability of the MTF-loaded LCS-ALG beads was 1.215 times higher compared to pure MTF, following oral administration of a single dose equivalent to 25 mg/kg body weight MTF to streptozotocin (STZ)-induced diabetic male Sprague-Dawley rats. Significant hypoglycemic effect was obtained for STZ-induced diabetic rats orally treated with MTF-loaded LCS-ALG beads compared to control diabetic rats. Over a period of 29 days, the STZ-induced diabetic rats treated with MTF-loaded LCS-ALG beads showed a decrease in the aspartate aminotransferase (AST), alanine aminotransferase (ALT), triglycerides, cholesterol, and low-density lipoprotein-cholesterol (LDL-C) levels, as well as an increase in glutathione peroxidase (GPx) and a recovery in the oxidative stress biomarker, lipid peroxidation (LPx). In addition, histopathological studies of liver, pancreas, kidney, and testes suggested that MTF-loaded LCS-ALG beads improved the degenerative changes in organs of diabetic rats. The LCS-ALG platform for dual encapsulation of MTF achieved sustained MTF delivery and enhancement of bioavailability, as well as the improved biochemical and histopathological characteristics in in vivo studies, opening many other intriguing applications in sustained drug delivery.

## 1. Introduction

The global figure of diabetic patients is projected to reach 700 million by 2045, where 90% of this figure is accounted for by patients with type 2 diabetes mellitus (T2DM) according to the International Diabetes Federation (IDF) [1]. The T2DM, a metabolic disorder characterized by insulin resistance and pancreatic *β*-cell disfunction [2,3], is considered a pandemic with no signs of mitigation [4]. Metformin (MTF), a biguanide antidiabetic agent, is well-established as a first-line therapy for the management of T2DM [5,6,7,8]. The well-established clinical benefits of MTF include antihyperglycemic efficacy, low toxicity, and negligible risk of hypoglycemia [7,9,10,11,12] without stimulating insulin secretion [13].

Moreover, MTF is frequently described as the “aspirin of the 21st century” [14], and some potential pleiotropic benefits of MTF have been recently emerged such as, inter alia, anti-cancer [15,16], anti-aging [17], anti-inflammation [10,18,19], and tackling the COVID-19 virus [20,21]. According to the Biopharmaceutics Classification System (BCS), MTF is classified as Class III high solubility–low permeability drug [22], where the hydrophilic base MTF (pKa 2.8 and 11.5) exists as cationic species at physiological pH [7,9]. The action of MTF involves the enhancement of insulin action by reducing hepatic gluconeogenesis and lipid production, yet its underlying mechanism is complicated and not entirely understood [10,13,23]. Despite being therapeutically efficacious and globally used for more than 60 years [5], there are still major concerns obstructing MTF extended efficacy, such as its limited oral bioavailability (~40–60%) due to its saturable and incomplete gastrointestinal (GI) tract absorption [7,24,25,26,27] and its short elimination half-life [12,28,29]. In addition, to reach MTF therapeutic efficacy, repeated high doses (2.5 g/day dosage) are frequently required, resulting in poor patient compliance, the occurrence of GI tract adverse effects, and the uncommon lactic acidosis incidences [30,31]. These concerns have stimulated researchers to explore novel formulation approaches in efforts to decrease MTF adverse effects and repeated dosing as well as to enhance its bioavailability and the patient compliance [11,13,32,33].

Recently, microencapsulation technology has been implemented as a potential approach to innovative drug delivery systems, providing promising improvements in targeted, sustained, and controlled delivery of the active pharmaceutical ingredients [32,33,34,35,36]. In addition, microparticles, owing to their small size, can spread out over the small intestine, resulting in improving drug absorption, decreasing dose-dumping risk, and allowing preprogrammed GI tract transit [11,35,37]. Several natural or synthetic polymeric materials have been investigated for microencapsulation of MTF [11,33,38,39,40,41,42,43]. Of these natural polymers, the polyanionic calcium alginate (ALG) biopolymer, which has been extensively investigated in interesting controlled drug delivery systems owing to its pH-sensitivity, biocompatibility, non-toxicity, and decent muco-adhesion properties [11,44,45]. Hydrogel microcapsules or beads of ALG can be easily prepared at room temperature using the ionotropic gelation technique, where a divalent cation, usually Ca^2+^, crosslinks the alginate chains via the “egg-box” model [46,47]. However, these microparticles or beads exhibited some concerns such as a fragile structure [48], the existence of surface wrinkles and cracks, and the accumulation of the loaded drug on their surfaces [45,49,50], even when the alginate was blended with other polymers during formulations [48,51].

**Scheme 1:**
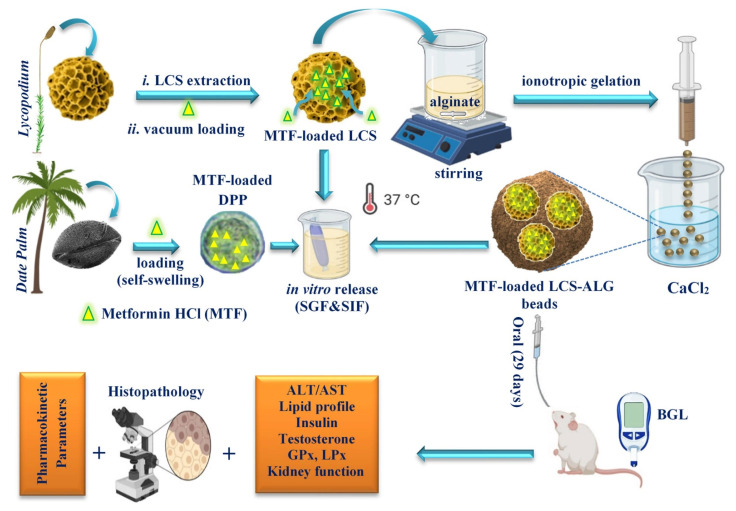
The protocol used for MTF encapsulation platform in the in vitro and in vitro studies.

Unlike the above-mentioned studies, we propose a different strategy for microencapsulation of MTF into plant-based pollen microcapsules and their secondary encapsulation into composite ALG beads. Here, we explore their feasibility in improving MTF sustainable and targeted in vitro and in vivo delivery, thus enhancing its pharmacokinetic properties and bioavailability. Natural sporopollenin microcapsules, the outer shells derived from raw pollens such as *Lycopodium clavatum,* have recently emerged as an alternative efficacious encapsulation platform for drugs [52,53,54,55], live cells [56], probiotics [57], pollen microgels [58], proteins [59], and vaccines [60]. Being natural biomaterials, these cost-effective sporopollenin microcapsules demonstrated several properties such as robustness, chemical resilience, unique surface ornaments, size uniformity, abundancy, and biocompatibility. The raw date palm (*Phoenix dactylifera* L.) pollens, DDP, are native to the Middle East region and have been extensively exploited in traditional herbal medicine since they contain some promising phytochemicals [61,62]. Our proposed protocol (Scheme 1) in the current study involves (i) extraction of LCS microcapsules from their raw spores and loading them with MTF via a vacuum technique; (ii) loading the raw DPP microcapsules with MTF using a novel hydration-induced swelling technique; (iii) double encapsulation of the MTF-loaded LCS microcapsules into ALG beads; (iv) evaluation of the in vitro MTF release profile of three formulations synthesized at different pH values; (v) in vivo study of the pharmacokinetic parameters and the glycemic control efficacy of the MTF-loaded LCS-ALG beads in STZ-induced diabetic rats; and (vi) evaluation of the biochemical and histopathological characteristics in the in vivo study.

## 2. Materials and Methods

### 2.1. Materials

Metformin hydrochloride was purchased from Pratap chemical industries Pvt. Ltd., Nagpur, India. Raw date palm, *Phoenix dactylifera *L., pollen powder was purchased from the Agricultural Research Centre, Giza, Egypt. *Lycopodium clavatum *L. spores (S-type), eosin Y, and Streptozotocin (STZ) were purchased from Sigma-Aldrich, Gillingham, Dorset, UK. Sodium hydroxide and potassium hydroxide were purchased from Fine Chemicals, Al-Gomhoria Company, Cairo, Egypt; liquid hydrochloric acid, calcium chloride, sodium chloride. and di-sodium hydrogen orthophosphate anhydrous were purchased from Adwic, El-Nasr Company, Cairo, Egypt; potassium di-hydrogen orthophosphate and potassium chloride were purchased from Alpha Chemika, Mumbai, India; and ethanol and methanol were purchased from Piochem, Giza, Egypt. Sodium alginate was purchased from BDH Laboratory Supplies, Poole, UK; dialysis membrane was purchased from Serva GmbH, molecular weight cut-off, MWCO: 12–14 kDa, diameter 21 mm, Heidelberg, Germany; and standard white clips (closed length 45 mm) were purchased from Carl Roth GmbH, Karlsruhe, Germany. Distilled water was used throughout all experiments.

### 2.2. Encapsulation of MTF into LCS Sporopollenin Microcapsules

Initially, LCS microcapsules, devoid of their sporoplasm materials, were extracted from their raw spores using a reported method [53,63]. Briefly, raw spores were successively treated with acetone to de-fat raw spores, then potassium hydroxide aqueous solution was used to remove genetic materials from the inner cavity, followed by a final orthophosphoric acid treatment to remove the polysaccharide intine. After each chemical treatment, spores were filtered through Whatman^®^ filter paper no. 1 (11 µm pore size), washed, dried at 60 °C until constant weight, and stored at room temperature until further use. Generally, loading the empty sporopollenin with active substances can be performed either by simple passive diffusion through the porous walls, or by vacuum-assisted loading and/or by compressing the encapsulant into tablets [53]. The vacuum-assisted loading route was used in the current work in which pure MTF:LCS (1:1, *wt*/*wt*) ratio was used, giving a theoretical drug loading (TDL) of 50%. First, 4.5 g of pure MTF crystalline powder was dissolved in 20 mL of methanol:water (1:1, *v*/*v*) solution in a small glass beaker and stirred for 5 min for complete dissolution. Then, 4.5 g of the dry empty LCS microcapsules were suspended in the MTF solution and stirred for 1 h at 500 rpm. The beaker was then placed in a vacuum desiccator at (ca. 20 mbar) for an additional 2 h at room temperature. The resulted MTF-loaded LCS microcapsules were collected by filtering through a Whatman^®^ filter paper 11 µm, washed with methanol:water (1:1, *v*/*v*) to remove any surface-adhered MTF, dried at 78–80 °C until constant weight, and eventually stored at room temperature until further use.

### 2.3. Encapsulation of MTF into Raw DPP Pollens

The loading of MTF into raw DPP pollens was carried out via the hydration-induced swelling route where the swelling of the raw dried oval-shaped pollens was noticed upon contacting with aqueous solutions only, which resulted in an instant swelling, producing nearly spherically shaped capsules. This interesting phenomenon will be discussed in detail in the next section. The loading processes were started by dissolving 200 mg of pure MTF in 2 mL of methanol:water (1:1, *v*/*v*) solution until complete dissolution of the MTF; then, 200 mg dried raw DPP was then added, and the suspension was allowed to mix for 1 h with stirring at 100 rpm at room temperature. This gives MTF:DPP (1:1, *wt*/*wt*) ratio, hence (TDL) of 50%. The MTF-loaded DPP microcapsules were then filtered using a Whatman^®^ filter paper 11 µm, washed with methanol:water (1:1, *v*/*v*) to remove any surface-adhered MTF, dried at 78–80 °C until a constant weight, and eventually stored at room temperature until further use.

### 2.4. Encapsulation of MTF-Loaded LCS into Calcium Alginate (ALG) Beads

To fabricate the MTF-loaded LCS-ALG composite microcapsules, a second level of microencapsulation of the prepared MTF-loaded LCS microcapsules was carried out utilizing the ionotropic gelation method, where sodium alginate was used as a polymer and calcium chloride as a cross-linker. Initially, 2% (*wt*/*v*) of sodium alginate dispersion was prepared in water at 60 °C with stirring at 500 rpm until complete dispersion. Then, the dried MTF-loaded LCS microcapsules were mixed with the sodium alginate dispersion for 10 min at 500 rpm, keeping the MTF-loaded LCS:sodium alginate ratio of (2.25:1, *wt*/*wt*), based on (TDL) of 40.91%. The resultant suspension was then quickly dropped into a freshly prepared 100 mL of 8% (*wt*/*v*) CaCl_2_ cross-linking bath using a 25 mL syringe fitted with a 23 gauge (G) hypodermic needle from a 5 cm distance above the CaCl_2_ bath surface with gentle shaking. The formed MTF-loaded LCS-ALG beads were completely cured in the CaCl_2_ bath by maintaining the stirring for 15 min at room temperature. Afterwards, the wet MTF-loaded LCS-ALG beads were filtered using a 11 µm Whatman^®^ filter paper, washed with 2 mL pure water twice to remove any excess of Ca^2+^ ions, allowed to dry at room temperature for 24 h, and eventually stored in brown vials in a dark condition at room temperature until further use.

### 2.5. Loading Capacity, Encapsulation Efficiency, and Production Yield

A total of 10 mg of the dry MTF-loaded LCS or MTF-loaded DPP microcapsules was suspended in 5 mL phosphate buffered saline (PBS, pH 7.4) and sonicated for 2 min in three cycles at 30% amplitude using an ultrasonic probe device (Ivymen^®^, CY-500, Barcelona, Spain) at room temperature. The suspension was then filtered, and 1 mL of the filtrate was diluted with PBS up to 10 mL and analyzed using the UV-VIS spectrophotometer (UV2600, Shimadzu, with UVProbe v.2.3 software for data acquisition and processing, Kyoto, Japan) at 233 nm against the appropriate blank. For the MTF-loaded LCS-ALG beads, the above procedures were followed except that the MTF-loaded LCS-ALG beads were kept in 5 mL PBS overnight before the sonication step to facilitate the disintegration of the calcium alginate. The amount of MTF was then calculated using the appropriate calibration curve, taking the dilution factor into consideration. The remaining solid microcapsules after filtration were resuspended in fresh PBS, subjected to further sonication cycles as mentioned above, and filtered to recover any remaining MTF. The last step was repeated two more times or until no MTF could be detected in the filtrate. Experiments were performed in triplicate, and the standard error of the mean (SE) was calculated from the mean values. The following equations were used to calculate the loading capacity (LC%), encapsulation efficiency (EE%), and production yield:

$$\mathrm{Loading}\;\mathrm{capacity}\;\left(\%\right)=\frac{\mathrm{amount}\;\mathrm{of}\;\mathrm{drug}\;\mathrm{entrapped}}{\mathrm{weight}\;\mathrm{of}\;\mathrm{drug}\;\mathrm{loaded}\;\mathrm{microcapsuls}}\times 100$$

$$\mathrm{Encapsulation}\;\mathrm{efficiency}\;\left(\%\right)=\frac{\mathrm{practical}\;\mathrm{drug}\;\mathrm{loading}\;}{\mathrm{theoretical}\;\mathrm{drug}\;\mathrm{loading}}\times 100$$

$$\mathrm{Production}\;\mathrm{yeild}\;\left(\%\right)=\frac{\mathrm{weight}\;\mathrm{of}\;\mathrm{dry}\;\mathrm{beads}}{\mathrm{initial}\;\mathrm{weight}\;\mathrm{of}\;\mathrm{MTF}\;+\;\mathrm{LCS}\;+\;\mathrm{ALG}}\times 100$$

### 2.6. In Vitro MTF Release from MTF-Loaded Microcapsules and Microbeads

A total of 10 mg of MTF-loaded LCS or 10 mg of MTF-loaded DDP sporopollenin or 20 mg of MTF-loaded LCS-ALG beads was dispersed into 3 mL of enzyme-free simulated gastric fluid (SGF, pH 1.2) solution in a dialysis bag (12–14 KDa MWCO), then placed with clips in a 60 mL SGF receptor solution thermostated at 37 ± 0.5 °C with fixed stirring at 100 rpm for the initial 2 h; then, the loaded microcapsules were filtered and transferred into a fresh 60 mL of the simulated intestinal fluid (SIF, pH 7.4) solution or additional 8 h, simulating the gastrointestinal (GI) tract conditions. At predetermined time intervals (0.25, 0.5, 1, 1.5, 2, 3, 3.5, 4, 5, 7, 8, and 9.5 h), aliquots of 4 mL were withdrawn from the release media and replenished by an equal volume of fresh SGF or SIF to keep the sink condition. MTF contents of collected samples, without dilution, were determined using the UV-VIS spectrophotometer by measuring the absorbance at 233 nm against the blank and applying the MTF calibration curve. Experiments were performed in triplicate and values expressed as mean ± SE.

### 2.7. In Vitro Drug Release Kinetics and Mechanism

To better understand and corelate the in vitro MTF release profile from the obtained different formulations, it is imperative to mathematically fit the release data to the known kinetic models (zero-order, first-order, and Higuchi) as well as the use of the Korsmeyer–Peppas model to ascribe the possible mechanism of drug release [64,65,66,67]. The zero-order kinetics model can be expressed by the following equation: *Qt* = *Q*_0_ + *K*_0_*t*, where *Qt* is the cumulative drug released at time *t*, *Q*_0_ is the initial amount of drug in the matrix, and *K*_0_ is the zero-order rate constant. The first-order kinetics model is described by the equation: *log C* = *log C*_0_−*K*_1_ *t*/2.303, where *C* is the percent of drug remaining at time *t*, *C*_0_ is the initial amount of drug in the solution, and *K*_1_ is the first-order rate constant. Higuchi’s model, based on the Fickian diffusion mechanism, is expressed by the simplified Higuchi equation: *Q_t_* = *K_H_ t*_1/2_, where *K_H_* is the Higuchi constant and *t* is the time. Korsmeyer-Peppas proposed a simple semi-imperial model, exponentially correlating the drug release to the time elapsed if no lag time or burst effect is present: *M_t_/M_∞_* = *K_kp_ t^n^ or log (M_t_/M_∞_)* = *log K_kp_ + n log*, where (*M_t_/M_∞_*) is the fractional release of the drug at time *t*, *M_t_* is the drug released at *t*, *M_∞_* is the drug released after time ∞, *n* is the drug release exponent, and *K_kp_* is the Korsmeyer release rate constant [64].

### 2.8. Optical, Fluorescence Microscopy and Digital Imagining

Optical images were taken by an Optika B-293 microscope, Ponteranica BG, Italy, fitted with Optikam, B5, 5MP digital camera. Images were recorded and processed using Optika IS view software. Digital images were captured using a portable digital microscope Plugable USB, 2MP, 250× magnification with adjusted LED halo light and graduated pad. Fluorescent images were recorded using an Olympus BX41 fluorescence microscope, fitted with a DP70 digital camera, Tokyo, Japan.

### 2.9. Scanning Electron Microscopy (SEM) and Transmission Electron Microscopy (TEM)

Scanning electron microscope analysis was acquired using JSM-5400 LV JEOL, Tokyo, Japan. Samples were coated with 20 nm gold using a gold sputter (JEOL JFC-1100E). An acceleration voltage of 15 kV was used during capturing at different magnifications. TEM images were obtained using a JEOL 2010 high resolution transmission electron microscope, Tokyo, Japan. DPP samples were treated with osmium tetroxide and sliced according to the reported method [68].

### 2.10. Fourier-Transform Infrared (FTIR) Spectroscopy Analysis

FTIR spectra were obtained using a Nicolet iS10 spectrometer, Thermo Scientific, Waltham, MA, USA. Samples were ground with anhydrous potassium bromide (Spectrosol^®^ grade) with a ratio of 1/9 (*w*/*w*) to obtain disks. All spectra were obtained in the range 4000–400 cm^−1^ with an average of 16 scan via scanning against a background, using OMNIC software.

### 2.11. Thermogravimetric Analysis (TGA)

TGA analysis was performed at a heating rate of 10 °C/min up to 700 °C in a flow of nitrogen at 20 mL/min using TGA-50H, SHIMADZU, Kyoto, Japan.

### 2.12. In Vivo Studies

#### 2.12.1. Animals

Clinically normal male Sprague-Dawley rats weighing approximately 150–200 g at the start of the experiment were used. The rats were obtained from the Laboratory Animal Centre (Dokki, Giza, Egypt). The rats were housed at a temperature of 25 ± 3 °C and were maintained under a natural daily 12 h light/dark cycle. They had unlimited access to a standard laboratory diet and tap water. The rats were left for at least one week prior to the experiment to acclimate. To reduce circadian effects, all experiments were carried out between 8:00 a.m. and 12:00 p.m. The study was conducted according to the guidelines of the Declaration of Helsinki, and the study protocol was approved by The Commission on the Ethics of Scientific Research, Faculty of Pharmacy, Minia University (Code number of the Project: ES01/2020, date of approval 29 October 2020). Every effort was made to minimize animal suffering and to reduce the number of animals used.

#### 2.12.2. Induction of Type 2 Diabetes Mellitus (T2DM)

T2DM was induced with a single intravenous injection of a freshly prepared solution of streptozotocin (STZ) 55 mg/kg [69] dissolved in a 0.01 M citrate buffer (pH 4.5) in rats subjected to 14 h fasting. The rats were allowed 5% glucose solution ad libitum overnight on the first day, post-STZ injection, to avoid possible hypoglycemic events and mortality due to the acute effect of STZ. Three days post-STZ-injection, the fasting blood glucose levels of the rats were measured from the tip of the tail using (GlucoDr SuperSensor, AGM-2200, with GlucoDr SuperSensor Test Strips, All Medicus Co. Ltd., Anyang-si, Korea). Animals with fasting blood glucose higher than 200 mg/dL were used for the study.

#### 2.12.3. Experimental Design

Thirty rats were randomly divided into five groups and dosed daily for 29 days according to the following scheme:

- Group 1: Normal control rats.
- Group 2: Vehicle group treated with LCS-ALG (250 mg/kg/day) dispersed in pure water.
- Group 3: Untreaded diabetic control group (Dia group).
- Group 4: Diabetic group treated with MTF (25 mg/kg/day) [70] dissolved in pure water (Dia + MTF group).
- Group 5: Diabetic group treated with MTF-loaded LCS-ALG beads (250 mg/kg/day), equivalent to 25 mg/kg bw MTF, dispersed in pure water (Dia+MTF-loaded LCS-ALG beads group).

All treatments were administered via oral gavage and all doses were adjusted each week based on changes in body weight to maintain an equivalent dose/kg/bw of rats for each group throughout the study duration.

##### Detection of MTF-Loaded LCS-ALG Beads in Rats’ Stomach

A preliminary test was performed to visualize the optimized MTF-loaded LCS-ALG beads in stomach of rats. In this test, MTF-loaded LCS-ALG beads were stained with 3 × 10^−3^ M Eosin Y, added to SA before gelling, to help detect them in stomach mucosa. Six normal male Sprague-Dawley rats weighing approximately 150–200 g were used, and each fasted rat was orally given a single dose (250 mg/kg bw) of the stained beads. After 2 h of administration, rats were sacrificed using diethyl ether. Rats were then dissected immediately after scarification. Afterwards, stomachs were removed, opened along the greater curvature, washed with normal saline, and examined using the portable USB digital microscope (described in Section 2.8).

#### 2.12.4. Body Weight

During the experiment, the body weight changes were monitored every week and at the end of the treatment period by an automatic GX-600 balance (A&D Company, Ltd., Tokyo, Japan).

#### 2.12.5. Blood Glucose Level (BGL)

Blood glucose levels were measured from the tip of the tail of animals for all groups at 0, 2, 4, 6, 8, and 10 h on Day 1 of the experiment. In addition, the BGLs were monitored weekly over 29 days for all groups at 7:00 a.m. using the glucometer. The percentage change and the percentage reduction (%) in BGLs were calculated relative to the measurement recorded after the T2DM induction period and before the treatments.

#### 2.12.6. High Performance Liquid Chromatography (HPLC) MTF Assay

Tail vein blood samples were taken from Group 4 (pure MTF) and Group 5 (MTF-loaded LCS-ALG beads) at 0, 1, 2, 4, 6, 8, and 10 h from the beginning of the experiment. Plasma samples were separated for MTF concentration determination. The HPLC assay for MTF was performed using Perkin Elmer (series 200) equipped with a variable wavelength UV detector and auto sampler, Waltham, MA, USA. A Phenomenex C18 symmetry (4.6 × 150 mm, 5 µm) column (Torrance, CA, USA) was used as the stationary phase at 25 °C. The mobile phase used was a (66:34, *v*/*v*) mixture of acetonitrile and phosphate-buffered saline (PBS) of pH 3, adjusted with orthophosphoric acid. A constant flow rate of 1 mL/min was used, and the volume injected was 20 mL and the MTF was detected at 235 nm. The average retention time of MTF was 9.61 ± 0.05 min. The limit of detection (LOD) and limit of quantification (LOQ) were 9.96 and 30.2 ng/mL, respectively. HPLC grade reagents were used for analysis.

#### 2.12.7. Pharmacokinetic Analysis

The pharmacokinetic (PK) parameters were calculated for the obtained MTF plasma concentration–time data using a Microsoft Excel add-in program (PKSolver) [71] employing the noncompartmental analysis method. The calculated PK parameters were the maximum observed plasma concentration (C_max_), time to reach C_max_ (T_max_), the half-life of the elimination phase (t_1/2_), the elimination rate constant (K_el_), the area under the plasma concentration–time curve from 0 to 10 h (AUC_0–10_), the area under the plasma concentration–time curve from time 0 to infinity (AUC_0–∞_), and the mean residence time (MRT_0–10_). The AUC was calculated using the linear-up/log-down trapezoidal method [71].

#### 2.12.8. Blood Sampling

At the end of the experiment (29 days), all animals were anesthetized using diethyl ether and euthanized by decapitation while fasting, and blood samples were collected from the trunk. Blood samples were centrifuged at 3000 rpm for 15 min to separate the serum. Furthermore, plasma samples were separated by centrifuging blood for 15 min at 3000 rpm with the anti-coagulant EDTA. Serum and plasma samples were stored at −80 °C for subsequent biochemical analysis.

#### 2.12.9. Biochemical Profile Assays

Serum alanine aminotransferase (ALT) and aspartate aminotransferase (AST) were measured using reagent kits purchased from the Spectrum Diagnostics kit. Total cholesterol (TC), high-density lipoprotein-cholesterol (HDL-C), low-density lipoprotein-cholesterol (LDL-C), and triglycerides (TGs) were measured in serum using Bio-Diagnostic kits. Serum creatinine, urea, and uric acid were measured using Diamond Diagnostics kits. Plasma lipid peroxide levels (LPx) and glutathione peroxidase (GPx) activities were determined calorimetrically using Bio-Diagnostic kits. Moreover, serum insulin and testosterone were measured using a rat-specific insulin and testosterone enzyme-linked immunoassay (ELISA) kits, respectively, from Crystal Chem. All experiments were performed according to the manufacturer’s instructions.

#### 2.12.10. Histological Procedures

For histopathological examination, the rats were dissected, and the liver, pancreas, kidney, and testes were instantly dissected out. The collected organs were fixed in 10% formal saline for 24 h before being processed with a graded ethanol series and embedded in paraffin. For optical microscopic examination, paraffin sections were cut into 5 µm thick slices using a microtome and stained with hematoxylin and eosin (H&E). Stained sections were examined for histological changes using light microscopy (Olympus CH20BIMF200, Olympus Optical Co. Ltd., Tokyo, Japan), and images were captured using a Micro Cam PHD-5MP camera, Lainsy Company, Cairo, Egypt. Stained tissue sections of the pancreas were examined under the light microscope for number of islets, and the islet area was determined in 10 islets per section at a magnification of ×100.

#### 2.12.11. Statistical Analyses

Data were analyzed using (SPSS version 22 for Windows). The Shapiro–Wilk test was used as the normality test. The significance was calculated using one-way analysis of variance (ANOVA) followed by Tukey’s multiple comparison procedure to calculate the significance. The data were expressed as the mean ± standard error of the mean (SE), and a *p*-value < 0.05 was considered as the level of significance.

## 3. Results and Discussion

### 3.1. Characterization of Raw Lycopodium Clavatum and Date Palm Pollens

**Figure 1:**
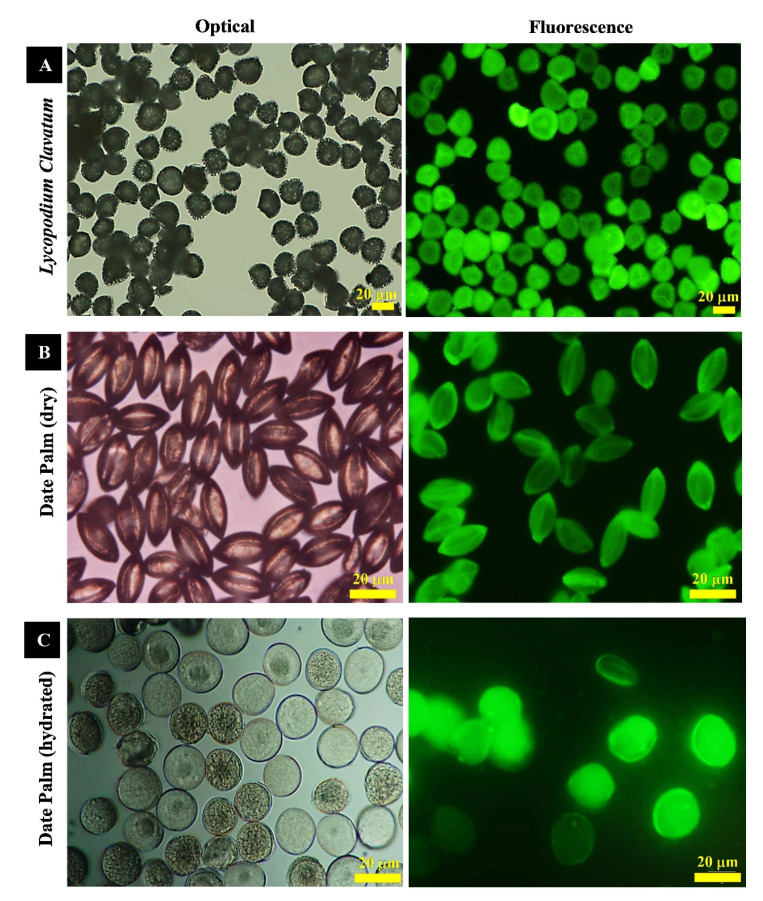
Optical and fluorescent images of raw *Lycopodium clavatum* and date palm pollens. (**A**) raw *Lycopodium clavatum* pollens, (**B**) dry date palm pollens, and (**C**) hydrated date palm pollens.

Plant pollens and spores protect male genetic materials from a harsh environment and transfer them to the female part of the flower during the pollination process. The typical microstructure of pollens comprises an inner shell layer (intine) that is composed mainly of cellulose and pectin, within which the sporoplasm is enclosed. The intine layer is protected by a robust outer shell (exine) predominantly composed of sporopollenin, a resilient biopolymer [52]. Figure 1A–C represents optical and fluorescent images of raw *Lycopodium clavatum* and date palm pollens. The uniformity in the size and the autofluorescence character of the two raw pollen species are evident. The average size of *Lycopodium clavatum* pollens was around 28 µm in diameter, whereas the size of DPP was 21 µm long and 11 µm wide, as revealed from the optical microscopy examination. Figure 1A shows that *Lycopodium clavatum* pollens have a globular shape, from the proximal view, whereas the DPP exhibited an elliptical shape, as seen in Figure 1B.

We have realized a unique natural phenomenon where the raw DPP pollens swell only when in contact with aqueous solutions, where their elliptical original shape in dry state changes to an almost spherical shape upon this fast hydration process, as shown in Figure 1C. Additionally, after drying the swelled-hydrated pollens, we noticed that the pollens attain their original oval shape again (see Movies 1 and 2, ESI). The median diameter of the swelled DPP in water calculated from the size distribution curve was 19.3 µm (Figure S1, ESI). This cycle of hydration (swelling) –dehydration (deswelling) of the raw pollens can be repeated for several rounds. This effect is known as the “Harmomegathic” or “Wodehouse” effect, where two different morphological situations occur in a pollen grain: the dry and hydrated states [72]. The exact mechanism of this effect is not fully known; however, it was attributed to the possible balancing between the need to keep the exchange between the grain and its environment and the necessity of minimizing the dehydration of the interior materials [73]. It was reported that the Harmomegathic effect is insignificant in rigid and thick-walled pollens [72].

**Figure 2:**
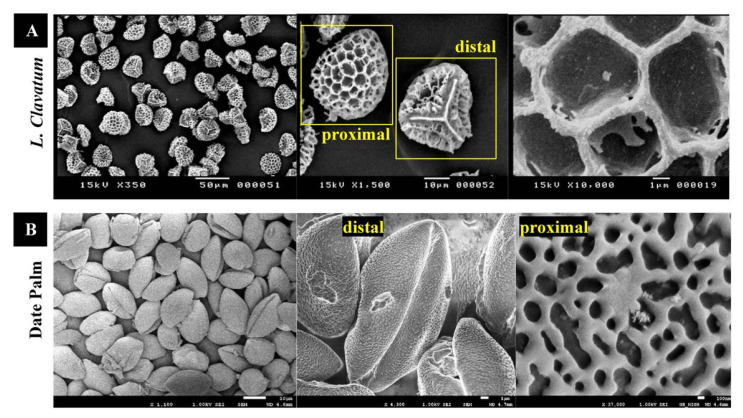
SEM images of raw pollens’ species showing their surface morphology at different magnifications. (**A**) *Lycopodium clavatum* and (**B**) date palm.

It was also observed that the spherical shape of the swelled DPP was retained permanently when they were dispersed in 10% CaCl_2_ or in cetyltrimethylammonium bromide (CTAB) cationic surfactant and then allowed to dry (Figure S2). Whilst the reason of such an effect has yet to be validated, it could be attributed to the formation of a sort of gelation structure within the pollen core that prevents them from folding back. We took advantage of these phenomena to explore the feasibility of encapsulating the positively charged MTF into the raw DPP via this novel hydration-induced swelling loading technique. The detailed surface morphology of the studied pollen can be better visualized using SEM, as shown in Figure 2A,B. The raw *Lycopodium clavatum* pollens are characterized by the reticulate ornamental elements as seen from the proximal view, while the trilete tetrad mark can be seen from the distal view, as shown in Figure 2A. As shown in Figure 2B and Figure S3, ESI, raw DPPs are featured by the perforate ornamental elements and a monolete aperture as seen from proximal and distal views, respectively. The aperture typically functions as the sole germination spot [72]. It was reported that the diverse surface decorative characteristics of sporopollenin could improve their muco-adhesion properties [60,74].

**Figure 3:**
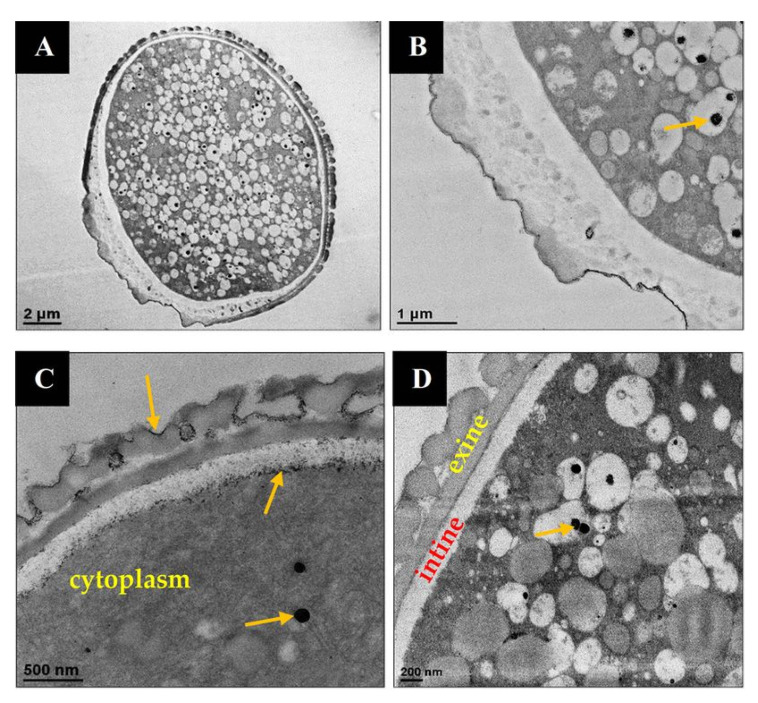
(**A**–**D**) TEM images of cross-section of a raw swelled DPP pollen showing its microstructure. Arrows indicate accumulation of staining materials on parts of intine and exine layers.

Figure 3A–D represents TEM images of a cross-section of a raw DPP showing its ultrastructure. The spherical-shaped swelled pollen and sporoplasm (enclosed within the intine layer) can be clearly seen in Figure 3A. The aperture region of pollen wall that lacks the exine ornamentations is the aperture membrane, which is usually characterized by a thick double-layered intine (Figure 3B). The thickness of exine layer was around 0.5 µm, as estimated from Figure 3, Figure S3 ESI. It was noticed that the osmium tetra-oxide, used in staining pollens, accumulated in the exine layer, inside the intine layer, and in dark spots within the cytoplasm mass, as is evident from the arrows in Figure 3C and energy-dispersive X-ray spectroscopy (EDX) spectra (Figure S4 ESI). This further supports our expectation that the swelled raw pollens, upon hydration, can transfer materials into their core beyond the intine layer. Various cytoplasmic organelles, such as vacuoles and starch granules, can be seen in Figure 3D.

Crucial for potential biomedical and drug delivery applications, the safety and biocompatibility of pollens and their extracted sporopollenin shells should be considered. It was reported that natural raw pollens of *Lycopodium clavatum* and date palm have been used in traditional herbal therapy since they contain promising phytochemicals [61,62,75]. In addition, it was shown that both raw pollens and their extracts, that constitute the allergens, did not cause oral hyposensitization in allergic subjects, suggesting the safety of raw pollens [55,76]. Interestingly, it was revealed that sporopollenin of *Lycopodium clavatum* formulated with ovalbumin, a model antigen, stimulated a “systemic and a mucosal immune response” following oral administration to mice [76]. Required for industrial applications, the biocompatibility and biodegradability of sporopollenin extracted from *Lycopodium clavatum* have been proven in several studies [52,53,56,74,77].

### 3.2. MTF-Loaded LCS, MTF-Loaded DPP Microcapsules, and MTF-Loaded LCS-ALG Beads

**Figure 4:**
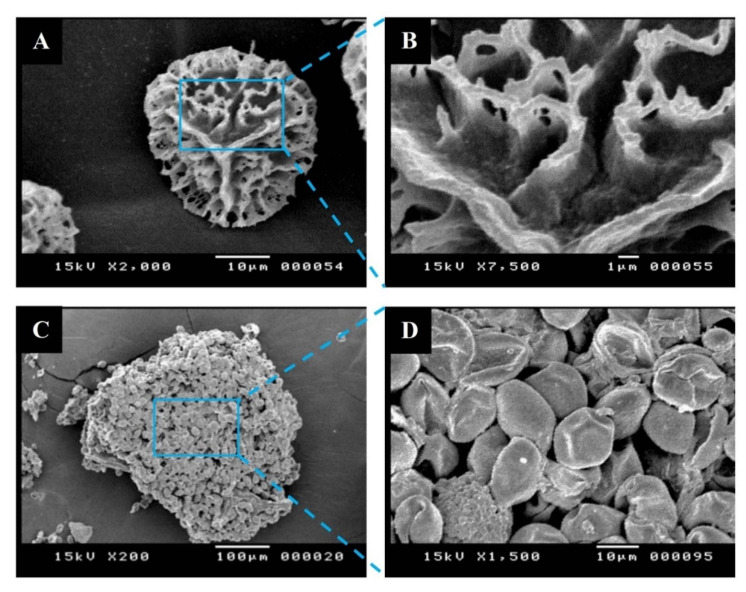
SEM images of microcapsules after MTF loading at different magnifications showing surface morphology. (**A**,**B**) MTF-Loaded LCS and (**C**,**D**) MTF-loaded DPP.

MTF has been loaded into the extracted empty LCS microcapsules via a vacuum protocol where no accumulation of MTF was noticed on the surface of the MTF-loaded LCS microcapsules, as is evident from the SEM images shown (Figure 4A,B). The possible route for MTF to cross the exine layer reaching the inner core of the empty LCS is through the nano multidirectional channels, responsible for in and out nutrition transport, in the exine layer [74]. It was reported that, for *Lycopodium clavatum* pollens, the diameter of the pores is around 3.2 nm [59]. For the raw DPPs, the MTF was loaded via the hydration-induced swelling of the DPPs upon contact with the aqueous methanolic solution of MTF. The SEM images of the MTF-loaded DPP are shown in Figure 4C,D, where the obtained microcapsules exhibited a tendency toward aggregation upon drying. In addition, MTF-loaded DPP microcapsules showed a swelled shape and did not return to their elliptical shape like the unloaded raw DPP. It is worth mentioning that suspensions of the obtained formulations in PBS did not show any aggregation tendency when examined under the optical microscope. The hydration-induced swelling loading technique offers a streamlined single-step loading process without using sophisticated equipment and excess chemicals. This could provide some advantages over the well-established microencapsulation techniques such as spray drying, coacervation, and freeze-drying that demand more sophisticated equipment, multi-step processes, and the use of high-priced excipients [36]. However, the limitation of the hydration-induced technique could be its strict use in aqueous solution of active ingredients. This drawback could be overcome via the solubilization of the hydrophobic ingredients into aqueous surfactant media. Being freely soluble in water and existing in a cationic form at the physiological pH range, it is reasonable to assume that the cationic MTF species could efficiently attach to the cytoplasmic matrix of the pollens where it was reported that the cell membrane of the cytoplasm is mainly anionic [78]. The %LC and %EE of both MTF-loaded LCS and MTF-loaded DPP are comparable and listed in Table 1.

It was imperative to examine the protection capability of the MTF-loaded LCS against the harsh stomach condition as well as to further target and sustain the release of MTF into the desired location. To explore this, the MTF-loaded LCS microcapsules were further encapsulated into ALG beads to form dual encapsulation composite platform for MTF. An 8% (*wt*/*v*) CaCl_2_ was used as a crosslinker for the ALG gelation process since it was reported that the degree of Ca^2+^-crosslinking increases at high concentrations of CaCl_2_ [45]. The ratio of MTF-loaded LCS:ALG was kept at (2.25:1, *wt*/*wt*) for the composite beads to minimize any potential interfering effects of ALG on the in vivo studies. In addition, MTF-loaded LCS was selected for ALG composite formation since the LCSs are devoid of any cytoplasmic materials, whereas the raw DPPs have several active ingredients that can have ameliorative effects on the selected biochemical parameters [61,62] in the in vivo studies of the current work. The production yield and the average diameter of the obtained MTF-loaded LCS-ALG beads were 74.3% and 700 ± 52 µm, respectively. The %LC, %EE, and production yield of the obtained formulations are listed in Table 1. It was crucial to achieve the best possible loading capacity with minimum treating processing. However, there is limited understanding of the underlying loading mechanisms of these natural microcapsules owing to the lack of literature dealing with their microencapsulation uses [79]. The high aqueous solubility of MTF and the low ALG content might have contributed to the low %LC for the obtained formulations. However, the obtained %LC values are comparable with those reported in other studies [79,80]. The %LC could be optimized by increasing the MTF:carrier wt.% ratio and adapting other encapsulation techniques such as ultrasonic power, freeze-drying, and centrifugal aided loading, which are currently under investigation.

**Figure 5:**
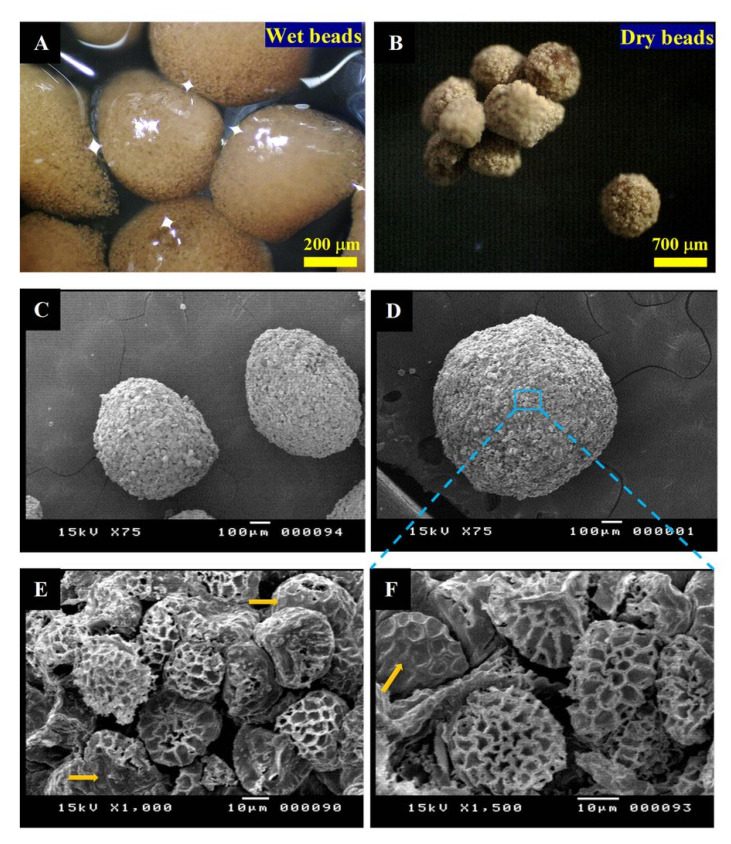
Optical and SEM images of MTF-loaded LCS-ALG beads showing the surface morphology. (**A**,**B**) Optical images of wet and dry beads. (**C**,**D**) SEM of beads showing their morphology and shape. (**E**,**F**) Surface of one bead at a higher magnification showing MTF-loaded LCS microcapsules with the ALG covering (arrows).

Figure 5A,B and Figure S5 ESI show optical images of the wet and dry beads, where the encapsulated MTF-loaded LCS microcapsules can be clearly seen to be impeded in the ALG polymer, reflecting the low ratio of the polymer used. Most of the beads were found to be nearly spherical in shape with a compact structure, and some beads appeared with an elongated tail, probably caused by the extrusion process. The surface morphology of the beads is shown in the SEM images in Figure 5C–F, where no visible surface cracks, wrinkles, or MTF accumulation were observed, indicative of the effectiveness of the protocol used. This is in contrast to previous studies where such surface issues were often reported in MTF-polymer beads [45,48] and microcapsules [49] due to the collapse of the polymer network upon drying. Figure 5E,F represents the beads’ surface at high magnifications, where the individual MTF-loaded LCS microcapsules can be clearly seen, some of them coated by the ALG polymer (indicated by arrows). Manual inspections of the beads revealed that they are relatively hard and can be cut easily by a spatula (more images are shown in Figure S6, ESI). It is worth mentioning that the optimized beads showed floating behavior when suspended in PBS during the loading capacity measurement; nevertheless, further validation is required. In addition, the LCS sporopollenin left after the multi-sonication process during the loading capacity test of the beads showed some damage to its ornamental features, as can be seen in Figure S7, ESI.

Despite providing several advantages, there are limited microencapsulated pharmaceutical products approved on the market since there are several challenges in their production from the standpoint of industry [34]. Of these challenges is the difficulty in achieving microparticles with size uniformity and well-defined surface morphology, resulting in poor reproducibility when scaling up the production [36]. It was proposed that the loading of sporopollenin microcapsules can be scaled up via vacuum-tumbling as an easier technique when compared to spray-drying. This was attributed to the salient features of natural pollens and sporopollenin, particularly their size uniformity, their well-defined surface ornaments for specific species, and the elasticity of their shells [74]. In line with that, these natural microcapsules could be compressed into tablet forms [56,74] with or without enteric coating [79] for safe administration in patients. In addition, microcapsules could be incorporated into various pharmaceutical dosage forms such as capsules, sachets, or semisolids (for topical application).

### 3.3. FTIR and Thermogravimetric Analyses (TGA)

To explore the possible interaction between the pure MTF and the LCS sporopollenin, DPP, and ALG polymer, the FTIR spectrum of the different formulations and pure substances was carried out and depicted (Figure S8, ESI). The pure MTF showed typical bands at 3368.21 and 3303.6 cm^−1^, assigned to (N−H) primary stretching vibration, whereas the band at 3161.37 cm^−1^ is due to (N−H) secondary stretching. The other characteristic bands for pure MTF, assigned for (C−N) stretching, were observed at 1624.8 and 1559.23 cm^−1^. The LCS showed a distinctive band at ~1513 cm^−1^ due to (C=C) stretching vibration of phenolic components of the sporopollenin [54]. For MTF-loaded LCS microcapsules, a sharp band appeared at 1559.23 cm^−1^ and a band at 1418.93 cm^−1^, both from MTF, confirming the encapsulation of MTF into the empty LCS. The spectrum of MTF-loaded DPP as seen in Figure S8, ESI, did not show significant changes compared to that of raw DPP apart from the appearance of a band of MTF at 1166 cm^−1^. Further analysis of FTIR data revealed the typical bands of sodium alginate powder at 1619.01 and 1408.32 cm^−1^, assigned to (COO^−^) asymmetric and symmetric stretching vibrations, respectively, where the latter band was shifted to 1431.95 cm^−1^ in the ALG beads spectrum as a result of the Ca^2+^ replacement of the uronic acid Na^+^ upon ionotropic gelation via the egg-box model [81]. In addition, the bands between 1150–1020 cm^−1^ for both SA powder and the ALG beads spectra were not significantly changed, suggesting that the glycosidic linkage remained unaffected upon gelation [81]. The spectrum of the optimized MTF-loaded LCS-ALG beads exhibited bands of both MTF-loaded LCS and the ALG without any significant shifts, suggesting that no significant interaction between MTF and the microcapsules or polymer used in the formulations.

TGA was performed under nitrogen, and its first derivative analysis to assess the thermal behavior of different formulations was obtained in this study, as shown in Figure S9A–C, ESI. All samples showed multistep degradations starting from the loss of absorbed water within the range of 33–200 °C. The TGA curve of the pure MTF showed very little loss in adsorbed water with around 2% weight loss before the start of the melting point of MTF (Figure S9A, ESI). We noticed an increase in weight loss of MTF-loaded microcapsule and bead composite when compared to unloaded microcapsules or ALG polymer beads, indicating that MTF was successfully loaded into these encapsulants (Figure S9A, ESI). The derivative TGA curve analysis allowed a better understanding of the degradation steps, as shown in (Figure S9B,C, ESI). For MTF-loaded LCS and MTF-loaded DPP microcapsules, the incorporation of MTF enhanced their thermal stability, as revealed by the increase in temperature of the maximum degradation rate of the second main peaks between 300–350 °C (Figure S9B,C, ESI). For MTF-loaded LCS-ALG beads, the temperature of the maximum degradation rate of pure ALG beads, ~299 °C, increased slightly to ~309 °C, indicating that the incorporation of ALG at a low ratio did not considerably enhance the thermal stability of composite beads. Generally, the thermal degradation steps of polysaccharides involve the loss of absorbed and crystalline water, the depolymerization of the polymer network, and eventually the formation of polynuclear aromatic carbon structures [82]. Moreover, it was reported that the degradation of cellulose, hemi-cellulose, and lignin accounts for the main mass loss for *Lycopodium clavatum* spores within a range of 250–550 °C [83].

### 3.4. In Vitro MTF Release Study

**Figure 6:**
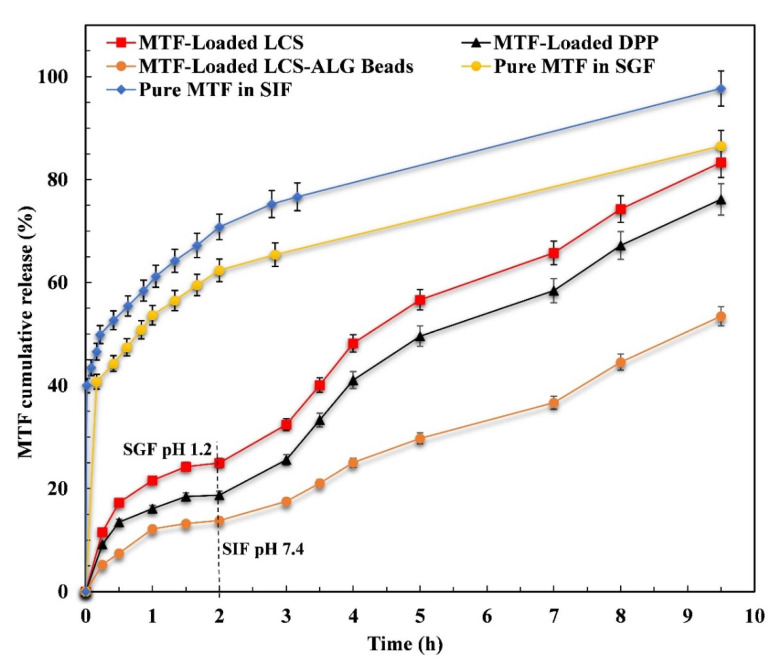
In vitro release profile of pure MTF and that released from MTF-loaded LCS, MTF-loaded DPP, and MTF-loaded LCS-ALG beads in different pH media at 37 ± 0.5 °C. Values are presented as mean ± SE, (*n* = 3).

We have studied the release profile of MTF in MTF-loaded LCS, MTF-loaded DPP, and MTF-loaded LCS-ALG beads to explore the feasibility of these encapsulation platforms in providing sustained and controlled MTF delivery. In addition, the release of pure MTF in SGF and SIF was conducted as a control for comparison purposes. The in vitro release of MTF from these formulations was carried out under SGF and SIF conditions, simulating the GI tract at 37 ± 0.5 °C as shown in Figure 6. For MTF-loaded LCS microcapsules, 25% of MTF was slowly released after 2 h in a gastric SGF condition, followed by a faster release in an intestinal SIF condition for the next 7.5 h reaching 84% release at 9.5 h. The release of MTF from MTF-loaded DPP was slower than that from MTF-loaded LCS where 19% was slowly released within 2 h in SGF and became faster in SIF, reaching 76% at 9.5 h. The dual encapsulation platform of MTF within the MTF-loaded LCS-ALG beads exhibited the slowest and most sustained MTF release compared to MTF-loaded LCS and MTF-loaded DPP microcapsules (Figure 6). In SGF, only 14% of MTF was slowly released from MTF-loaded LCS-ALG beads within 2 h, followed by faster release in SIF reaching 54% at 9.5 h. The release of pure MTF was much faster when compared to that released from encapsulated formulations, where 62.35% and 70.79% were released after 2 h in SGF and SIF, respectively, reflecting the high solubility of pure MTF that readily diffused through the dialysis membrane.

In all cases, release profiles shown in Figure 6 were pH-dependent and revealed a biphasic character staring with an initial small burst effect, a plateau in SGI within 2 h, followed by a sustained release phase over 7.5 h in SIF. The initial burst effect could be due to the adsorbed MTF on the surface of LCS-ALG bead formulations. For the MTF-loaded LCS-ALG beads, the slowest release of MTF in SGF compared to other formulations could be attributed to the dual encapsulation platform used, where MTF needs to be transported through two layers: the LCS shells where it was initially encapsulated and the second ALG polymer that covered the LCS microcapsules. In addition, it was shown that, for ALG in SGI, water-insoluble alginic acid is formed as a result of the protonation of the carboxylate groups of ALG polymer, resulting in limited swelling of the ALG that in turn impeded the release of the drug [38,49]. The situation was different in SIF where the ionotropically gelled ALG was found to swell and allowed faster drug release as a result of electrostatic repulsion between polymer chains in alkaline media, as reported in other studies [38,49,84]. Therefore, the optimized MTF-loaded LCS-ALG beads were selected as a composite platform for a potential intestinal-targeted MTF release in the next in vivo study.

Controlling the drug release rate and extent enable pharmacists and engineers to formulate controlled drug delivery systems to sustain action of the therapeutic agents. Therefore, to better understand the competitive MTF release mechanisms, various kinetic models were fitted to the experimental data obtained in Figure 6, and the results were summarized in Table S1, ESI. The highest correlation coefficient (*R*^2^) defines the most probable model of drug release kinetics and shows that the type of diffusion mechanism can be ascribed using the Korsmeyer–Peppas semi-mathematical model [64]. According to this model, for spherical geometry, the exponent *n* = 0.43 for Fickian diffusion, 0.43 < *n* < 0.85 for anomalous transport or non-Fickian diffusion, and *n* > 0.85 indicates a super case II transport mechanism controlled by polymer swelling and relaxation [64]. The release of pure MTF in both SGF and SIF media followed first-order kinetics with Fickian diffusion (Table S1, ESI). It was revealed that MTF release from the MTF-loaded LCS microcapsules followed the Higuchi model in both SGF and SIF media, indicating that the release kinetic followed the diffusion mechanism [85]. The exponent *n* for MTF-loaded LCS in SGI indicated Fickian diffusion of MTF, whereas in SIF, a non-Fickian diffusion can be assigned. Further inspection of data shown in Table S1 (ESI) revealed that MTF-loaded DPP microcapsules exhibited a similar kinetic and release mechanism pattern in SGF to MTF-loaded LCS. However, MTF release from MTF-loaded DPP in SIF followed first-order kinetics, a concentration-dependent release, with a super case II transport mechanism. Regarding the release of MTF from optimized MTF-loaded LCS-ALG beads, the Fickian diffusion mechanism in SGF was ascribed, whereas in SIF, the release followed zero-order kinetics, indicating a controlled release pattern with a super case II transport mechanism.

### 3.5. In Vivo Study

#### 3.5.1. Pharmacokinetics (PK) Evaluation

**Figure 7:**
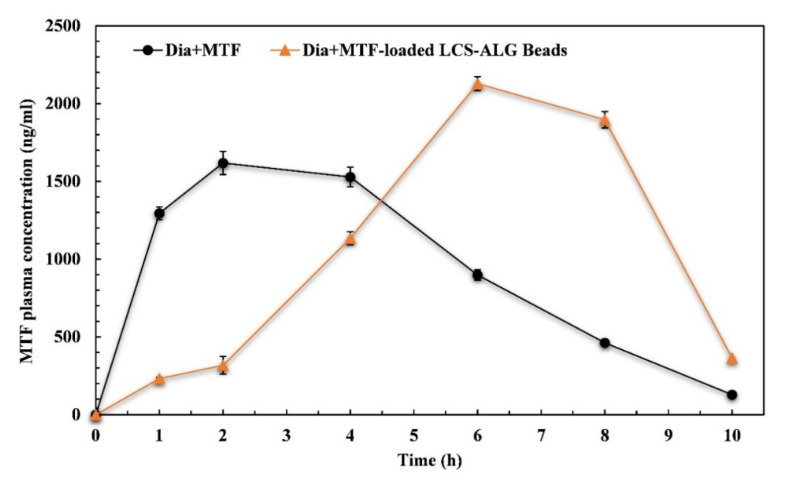
Mean plasma MTF concentration-time profile for pure MTF and MTF-loaded LCS-ALG beads following single oral administration equivalent to 25 mg/kg bw of MTF to STZ-induced diabetic Sprague-Dawley rats. Data shown as mean ± SE, (*n* = 6). Solid lines are guides to the eye.

The plasma MTF concentration–time profile after oral administration of pure MTF and optimized MTF-loaded LCS-ALG beads is shown in Figure 7, and the calculated PK parameters are summarized in Table 2. The optimized MTF-loaded LCS-ALG beads showed a significant (*p* < 0.05) higher maximal MTF concentration (C_max_ = 2128.5 ng/mL) when compared to that of pure MTF (C_max_ = 1618 ng/mL). The PK parameters also showed no significant difference for t_1/2_ between the pure MTF and MTF-loaded LCS-ALG beads, whereas the latter showed significantly higher T_max_ (6 h) compared to that of the former (2 h), indicating that MTF absorption was delayed when released from optimized beads. In addition, at 6 h, MTF level reached 2128.5 ng/mL for MTF-loaded LCS-ALG beads while it sharply declined to 898.5 ng/mL for pure MTF, which further confirms delayed MTF absorption for optimized beads. The data shown in Table 2 revealed that the relative bioavailability of MTF delivered from optimized MTF-loaded LCS-ALG beads was 121.5% in comparison to pure MTF. Therefore, the obtained data suggested that optimized MTF-loaded LCS-ALG beads improved MTF bioavailability. In addition, the mean residence time (MRT_0–10_ = 6.13 h) for optimized MTF-loaded LCS-ALG beads formulation was increased significantly compared to that of pure MTF (MRT_0–10_ = 3.97 h), indicative of a possible extended residence time of MTF at the absorption site [86]. The reported mucoadhesion of ALG polymer, coupled with the potential mucoadhesion ability of LCS sporopollenin microcapsules [60,74], might have contributed to the increase in (MRT_0–10_) of MTF released from beads.

**Figure 8:**
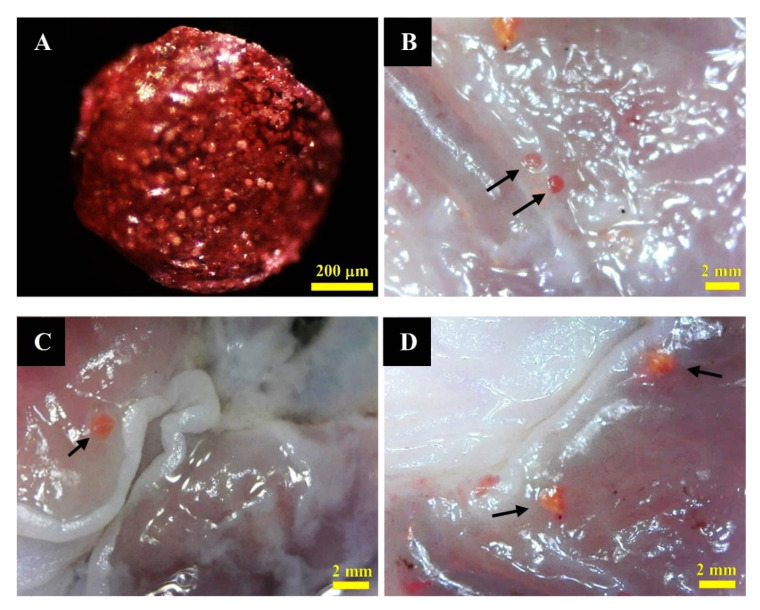
Optical and digital images of eosin Y stained MTF-Loaded LCS-ALG beads following oral administration to normal Sprague-Dawley rats. (**A**) optical image of a stained bead showing the encapsulated MTF-loaded LCS embedded inside. (**B**–**D**) Beads seen in stomach mucosa of the rats after 2 h of administration.

The results obtained from the PK evaluation shown in Figure 7 and Table 2 are consistent with the in vitro sustained release results of MTF in the SIF medium from the optimized beads in Figure 8. In line with that, it was reported that the absorption of MTF is mainly achieved at the proximal small intestine (duodenum and jejunum), whereas limited absorption is expected in the stomach and large intestine [7,26,29]. In addition, it has been shown that the intestinal absorptivity of MTF is site- and concentration-dependent, and about 40% of the orally administered dose is excreted in feces, indicative of incomplete absorption [7,87]. It was reported that, for MTF immediate release (IR) tablets, a lower single dose of 500 mg showed higher bioavailability compared to a higher dose of 2550 mg, which was attributed to a possible decrease in MTF absorption rather than a variation in its elimination [88]. Therefore, the optimized MTF-loaded LCS-ALG beads obtained by the dual encapsulation platform can offer a potential site-specific MTF sustained delivery system. Moreover, the obtained PK parameters for the optimized formula in the current study could be considered at a competitive level with that reported for MTF immediate-release (IR) or extended-release (XR) formulations [7,11,89,90].

#### 3.5.2. Detection of MTF-Loaded LCS-ALG Beads in Rats’ Stomach

Figure 8A shows a single stained bead where the embedded MTF-loaded LCS microcapsules can be seen. After 2 h of administration and washing the stomach with normal saline, few beads were seen embedded within stomach mucosa as indicated by arrows in Figure 8B–D in different parts of the stomach. It was reported that prolonged retention of the drug in the stomach by using gastroprotective and mucoadhesive formulations is beneficial for enhancing the drug bioavailability and reducing the dosing frequency [86]. Therefore, our preliminary results shown in Figure 8A–D suggest that the optimized beads show promising mucoadhesion properties; however, more comprehensive experiments are required to validate these potential properties.

#### 3.5.3. Changes in Body Weight

When compared to other groups, the Dia rat group showed a significant decrease in body weight at 14, 22, and 29 days from the beginning of the experiment. At the end of the experiment, however, there was a significant increase in body weight in the MTF and MTF-loaded LCS-ALG bead groups compared to the Dia rats. Administration of LCS + ALG to untreated rats did not result in any notable change in the body weight over a 29-day period when compared to the control rats (Figure S10, ESI). Shirwaikar et al. [91] reported that hyperglycemia is associated with increased muscle protein destruction, resulting in a reduction in animal weight. Additionally, the decrease in insulin secretion, which is one of the most anabolic hormones in the body, could explain the body weight loss [61]. Diabetic rats treated with MTF and MTF-loaded LCS-ALG beads gained significantly more body weight than Dia rats, possibly due to their protective impact on controlling muscle wasting, such as reversal of gluconeogenesis.

#### 3.5.4. Blood Glucose Level (BGL)

**Figure 9:**
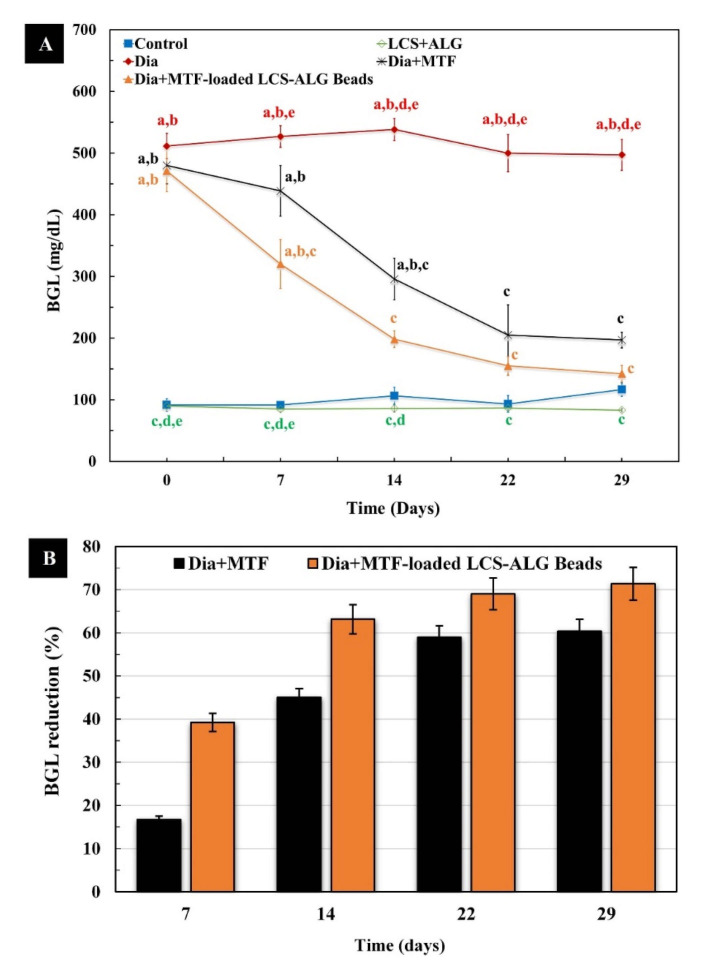
(**A**) Changes in blood glucose level, BGL (mg/dL), during the treatment period in the different studied groups. Data are presented as (mean ± SE, *n* = 6), significance at *p* < 0.05, a: significantly different when compared to control, b: significantly different when compared to LCS + ALG, c: significantly different when compared to Dia, d: significantly different when compared to Dia + MTF, e: significantly different when compared to MTF-loaded LCS-ALG bead group. (**B**) Percentage of reduction in BGLs of Dia + MTF and Dia + MTF-loaded LCS-ALG beads over 29 days. Solid lines are guides for the eye.

In the present study, Dia rats showed significantly (*p* < 0.05) higher fasting BGL compared to other groups. Interestingly, Dia rats treated with MTF-loaded LCS-ALG beads showed maintenance of the glycemia values after 14 days of treatment (Figure 9A). The reduction percentages of BGLs at Day 7, 14, 22, and 29 were 16.74, 45.04, 59, and 60.37, respectively, in MTF treated group, whereas reduction percentages of BGLs in MTF-Loaded LCS-ALG beads at Day 7, 14, 22, and 29 were 39.23, 63.15, 69, and 71.35, respectively, revealing the beneficial effects of the examined beads on glycemic control (Figure 9B).

MTF’s most well-known effect is the reduction of hyperglycemia, which could be attributed to inhibited hepatic gluconeogenesis, resulting in decreased hepatic glucose output [92]. MTF reduced the hepatic glucose production in rats by 50% to 60%, according to Song et al. [93]. Another important impact of MTF, associated with maintenance of glycemia, is the increase in insulin signaling, which leads to an increase in skeletal myocyte glucose uptake [94,95]. Additionally, several studies have recently suggested that MTF increases glucose intestinal absorption and utilization in enterocytes, limiting glucose access to the bloodstream [96,97]. The current study indicated that MTF-loaded LCS-ALG bead treatment had a better glucose-lowering effect relative to pure MTF during the treatment period. We hypothesized that, when compared to pure MTF, the beads were able to maintain the drug in an effective way as well as maintain the blood glucose level for a longer period.

**Figure 10:**
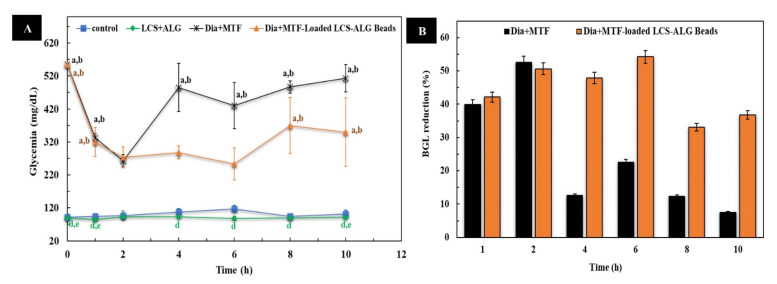
(**A**) Changes in blood glucose level (BGL) over 10 h on Day 1 of the experiment. Data are presented as (mean ± SE, *n* = 6), significance at *p* < 0.05. a: significantly different when compared to control, b: significantly different when compared to LCS + ALG, d: significantly different when compared to Dia + MTF and e: significantly different when compared to MTF-Loaded LCS-ALG bead group. (**B**) Percentage of reduction in BGLs of Dia +MTF and Dia + MTF-loaded LCS-ALG bead groups over 10 h.

The BGLs and the mean % reduction in BGLs obtained after administration of MTF and MTF-loaded LCS-ALG beads over 10 h are displayed in Figure 10A,B, respectively. The group that received pure MTF encountered a relatively rapid decrease in BGLs, reaching the minimum value after about 2 h with a 52.58% reduction in BGL, then rapidly returning to the baseline with a 7.56% reduction at 10 h. The decline in BGLs was slower in the MTF-loaded LCS-ALG bead group, and the reduction in BGLs reached its maximum of 54.21% at 6 h. Surprisingly, the BGLs did not return to the baseline after 10 h, with a 36.76% reduction. Nayak et al. [45] reported that fenugreek seed mucilage–alginate mucoadhesive beads containing MTF had a significant hypoglycemic effect, with a decrease in BGL of more than 25% in 1–2 h and a decrease of more than 30% in BGL over 10 h. Our formulation showed a better reduction, where the sustained hypoglycemic effect in MTF-loaded LCS-ALG beads was observed for longer period with more reduction percentages in BGLs throughout the experimental period.

#### 3.5.5. Biochemical Studies

##### Lipid Profile

**Figure 11:**
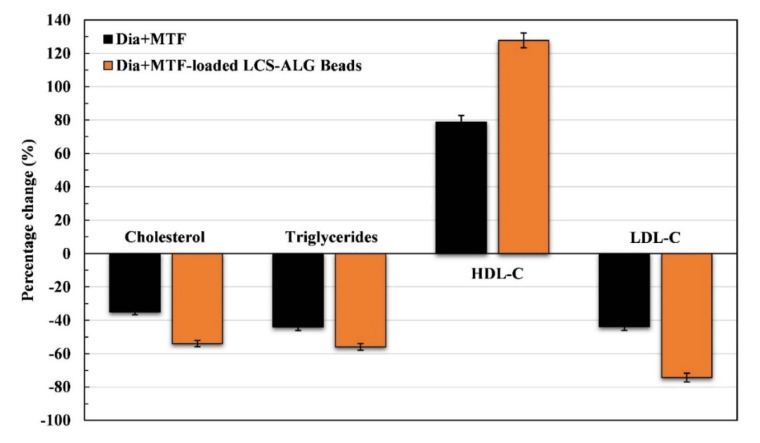
Percentage changes in lipid profile examined in Dia + MTF and MTF-loaded LCS-ALG bead groups relative to the Dia group at the end of the treatment period (29 days).

As shown in Figure S11A–D, ESI, there were no significant alterations in serum lipid profile in the group treated with LCS + ALG (Group 2) compared to the normal control group (Group 1). However, TC, TGs, LDL-C, and HDL-C levels in the Dia group showed significant changes when compared to other groups (*p* < 0.05). In addition, the administration of MET (Group 4) precluded the increase in TC, TGs, and LDL-C levels as well as the significant decrease in HDL-C compared to the Dia group. The TC, TGs, and LDL-C levels decreased to 35%, 44%, and 43.92%, respectively, but the HDL-C level increased to 78.86% in the MET group compared to the Dia rats (Figure 11). The MTF-Loaded LCS-ALG beads significantly corrected the serum lipid profile in Group 5 when compared to the Dia group (*p* < 0.05). Noticeably, nearly complete normalization of TGs and LDL-C values was observed in Group 5, and the percentages of decrease in TC, TGs, and LDL-C were 54%, 56%, and 74.33%, correspondingly, while the percentage of increase in HDL-C was 127.75% in the MTF-loaded LCS-ALG bead group compared to the Dia group (see Figure 11).

Regarding our results, the Dia group showed a significant increase in serum lipid profile. This is consistent with the results of Shao et al. [98], who revealed that disturbed lipid metabolism in diabetes mellitus (DM) has been well-recognized both clinically and experimentally. Additionally, it has been reported that hyperglycemia in DM, combined with the lack of insulin sensitivity, resulted in fatty acid mobilization from adipose tissues and proteins, leading to an increase in free fatty acids [99]. Latterly, hyperglycemia has been linked with disorders, which are typically characterized by hypertriglyceridemia combined with a low level of HDL-C. [100].

The treatment of Dia rats with MTF partially restored the dyslipidemia. Likewise, previous studies [101] reported that individual and concomitant treatments of sitagliptin (a dipeptidyl peptidase-4 inhibitor) and MTF remarkably corrected the level of TC and TGs compared to untreated diabetic rats. Similarly, Ismail et al. [102] reported that MTF was observed to normalize insulin-resistance-associated dyslipidemia along with regulating the expression of various genes associated with lipid metabolism. Interestingly, the effects of MTF on lipid profile have been thoroughly defined [103].

In comparison to MTF treatment alone, the MTF-loaded LCS-ALG bead group showed a considerable improvement in lipid profile. We suggested that the lipid-lowering effect of MTF-loaded LCS-ALG beads in animals studied might be due to their formulation, which prolonged the improvement effect of MTF on lipoprotein metabolism and increased the insulin sensitivity of adipose tissue by inhibiting the lipolysis and reducing the release of free fatty acids from adipocytes and their accumulation in the liver and other organs [12].

##### Liver Enzymes (ALT and AST)

**Figure 12:**
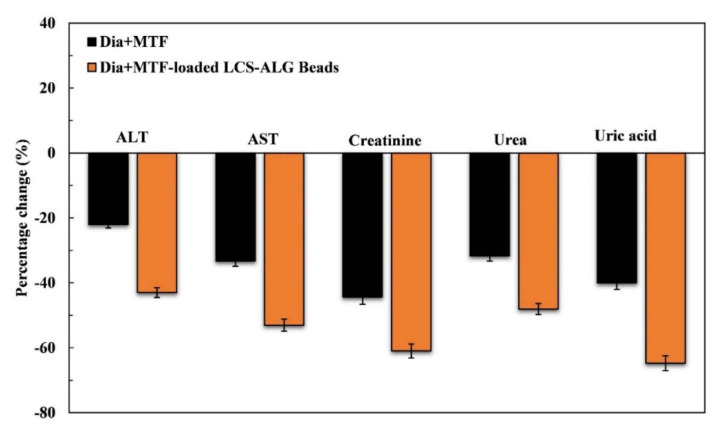
Percentage changes in the biochemical parameters examined in Dia + MTF and MTF-loaded LCS-ALG bead groups relative to the Dia group at the end of the treatment period (29 days).

As shown in (Figure S12A,B, ESI), all Dia groups showed a significant increase (*p* < 0.05) in serum ALT and AST compared to all experimental groups. In the Dia + MTF group, ALT and AST levels were decreased significantly compared to the Dia group (Group 3) with percentages of decrease of 22% and 33.24%, correspondingly (Figure 12). Additionally, ALT and AST levels in the MTF-loaded LCS-ALG bead group were significantly lower than those obtained in the Dia group, and the percentages of decrease were 43% and 53.04%, respectively (Figure 12). The results of the current study revealed a significant difference in serum levels of ALT and AST in all treated groups when compared to control and LCS + ALG animals. The presence of elevated levels of ALT and AST in the serum of STZ-diabetic animals reflects impaired liver function. Such elevation is a well-known effect of T2DM, as reported in the previous clinical studies [104,105,106]. For the Dia group treated with MTF (Group 4), the analysis showed a significant decrease in ALT and AST compared to the Dia group, although not reaching the control levels, indicating liver recovery and homeostasis commencement [107]. Furthermore, Kabil et al. [108] reported that the protective activity of MTF might be due to its effect against cellular leakage and loss of functional integrity of the cell membrane in hepatocytes. Therefore, MTF-loaded LCS-ALG beads considerably improved the examined liver enzymes compared to the MTF-treated group.

##### Kidney Function

Following the induction of hyperglycemia via STZ, significant increases in serum creatinine, urea, and uric acid levels were detected as compared to the other experimental rats (Figure S12C–E, ESI). Notably, Dia rats treated with MTF alone showed a significant improvement (*p* < 0.05) in kidney parameters in comparison to the Dia group. The percentages of decrease in creatinine, urea, and uric acid were 44.35%, 31.68%, and 40.04%, respectively (Figure 12). Interestingly, MTF-loaded LCS-ALG beads have nearly normalized the values where percentages of decrease in creatinine, urea, and uric acid were 60.97%, 48.07%, and 64.76%, respectively, when compared to the Dia group (Figure 12).

Kidney dysfunction is well-known in diabetic animals and humans, where hyperglycemia causes chronic inflammation and tissue injury by increasing oxidative stress and the generation of free radicals [109], consistent with the current study. MTF appears to have nephroprotective properties, attenuating kidney injury caused by various toxins, including exposure to high glucose concentrations [110]. Several studies suggested that MTF may reduce kidney damage by inhibiting the accumulation of advanced glycation end products in various tissues and decreasing inflammation and oxidative stress in hyperglycemia [111,112]. Additionally, MTF increases the level of an anti-aging protein (klotho protein) in mammalian cells, which may slow down the progression of various kidney diseases [10]. To this end, it can be suggested that the MTF-loaded LCS-ALG beads showed superior nephroprotective effects when compared to MTF alone.

##### Testosterone Level

**Figure 13:**
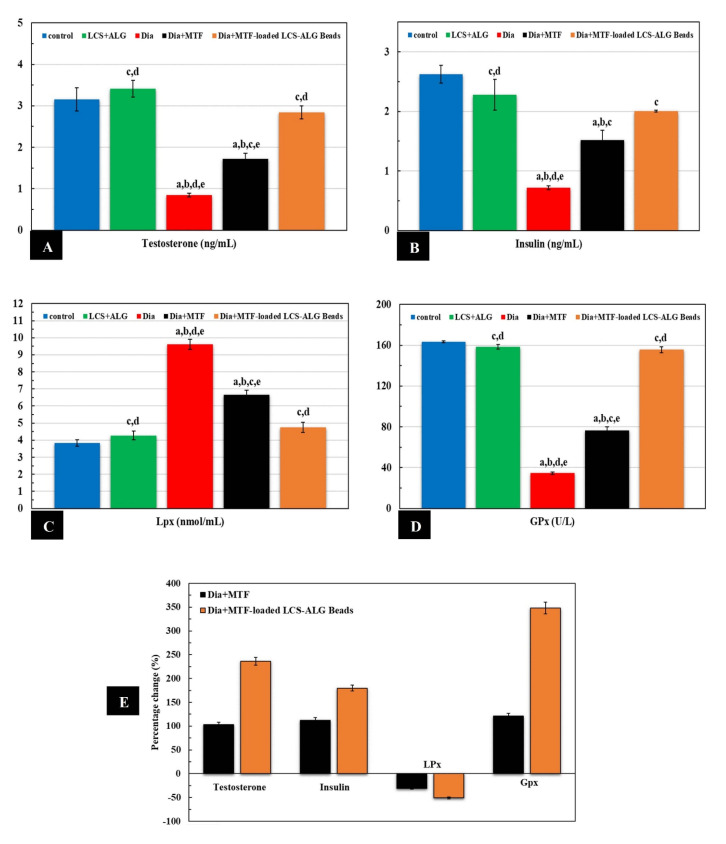
(**A**) Levels of testosterone, (**B**) insulin, (**C**) LPx, and (**D**) GPx at the end of the treatment period (29 days). Data are presented as (mean ± SD, *n* = 6), significance at *p* < 0.05. ^a^: significantly different when compared to control, ^b^: significantly different when compared to LCS + ALG, ^c^: significantly different when compared to Dia, ^d^: significantly different when compared to compared to Dia + MTF and ^e^: significantly different when compared to MTF-loaded LCS-ALG bead group. (**E**) Percentage changes in biochemical parameters examined in Dia + MTF and MTF-loaded LCS-ALG bead groups relative to Dia group.

The serum testosterone levels of different groups are shown in (Figure 13A). The Dia group showed a significant decrease in serum testosterone concentration (*p* < 0.05) when compared to other groups. However, the testosterone levels increased (*p* < 0.05) following the administration of MTF and MTF-loaded LCS-ALG beads relative to the Dia group. When compared to the Dia rats, the percentages of increase in MTF and MTF-loaded LCS-ALG beads were 103% and 236%, correspondingly (Figure 13E). In the current study, testosterone levels dropped significantly in the Dia group. These findings were consistent with previous studies [113,114], which found lower serum testosterone levels in experimental rats and concluded that the androgen synthesis may be suppressed. Furthermore, the decrease in activities of 3β-hydroxysteroid dehydrogenase (HSD) and 17β-HSD enzymes was concerned with downstream reactions in steroidogenesis in testis of experimental rats, explaining the lessened testosterone level in the Dia group. The MTF-treated group increased testosterone level significantly more than of the Dia group, with an interestingly better response in MTF-loaded LCS-ALG bead-treated rats. Several studies [115,116] found that treating male rats with severe T2DM with MTF preserves their androgenic status, owing to adjusted steroidogenesis along with the increased testicular levels of enzymes 3β-and 17β-HSDs and cytochrome P450 17A1 (CYP17A1). Furthermore, the increased insulin levels reported in the current study may play a role in the recovery of the Leydig cell physiology, which in turn causes an increase in testosterone [117]. It was also reported that MTF shows a significant influence on energy metabolism and signal transduction in the testicular steroidogenic as well as generative cells [116].

##### Insulin Level

By the end of the treatment period, all Dia rats showed a significant decrease (*p* < 0.05) in serum insulin compared to other experimental groups (Figure 13B). MTF-treated rats showed a significant increase in serum insulin levels when compared to the Dia group (*p* < 0.05) with percentage of increase of 112%, while MTF-loaded LCS-ALG bead-treated rats showed the highest percentage of increase of 180% compared to the Dia group (Figure 13E). Gregg et al. [118] reported that MTF raises the plasma level of glucagon-like peptide 1 (GLP-1) and stimulates the function of GLP-1 on pancreatic β-cells, thus increasing insulin secretion. In the same line, Ismail et al. [102] concluded that MTF treatment was shown to stimulate pancreatic β-cell regeneration and positive insulin signaling. Additionally, it should be observed that the MTF’s direct effect on insulin resistance possibly allows a wider medicinal usage [119]. Approximately, MTF-loaded LCS-ALG bead treatment in Group 5 induced the insulin value standardization, implying a special functionality and specific feature of MTF formulated in the examined beads.

##### Oxidative Stress

Figure 13C,D show that LPx and GPx values significantly increased and decreased, respectively (*p* < 0.05), in the Dia group compared to all groups, whereas the results of the control and LCS + ALG treatments showed no significant differences. However, in MTF-treated animals (Group 4), both parameters were significantly maintained (*p* < 0.05) compared to the Dia rats and percentages of the decrease in LPx, and the increases in GPx were 31% and 121%, correspondingly (Figure 13E). The MTF-loaded LCS-ALG bead group showed approximate normalization of LPx and GPx values when compared to control and LCS + ALG animals. Additionally, the LPx levels decreased to 51%, but the GPx levels increased to 348% compared to the Dia group (Figure 13E).

Diabetes causes high oxidative stress in both human and experimental animal studies, which decreases the activity of free radical scavenging enzymes and hence enhances the free radical generation [120,121]. In agreement with the results of the present investigation, many studies reported that MTF alleviates oxidative stress and improves antioxidant status [122]. Consistent with this concept, Faure et al. [123] reported that MTF causes an increase in the reduced glutathione. The previous findings are supported by Singh et al. [124], who reported a significant decrease in malondialdehyde (MDA) levels and an increase in superoxide dismutase (SOD), antioxidant defense system, in MTF-treated animals. All diabetic groups treated with MTF-loaded LCS-ALG beads further reduced their oxidative stress compared to the MTF individually treated group. The findings presented herein supported our hypothesis regarding the potential therapeutic application of MTF loaded into the optimized formula of LCS-ALG beads.

#### 3.5.6. Histological Studies

##### Liver

**Figure 14:**
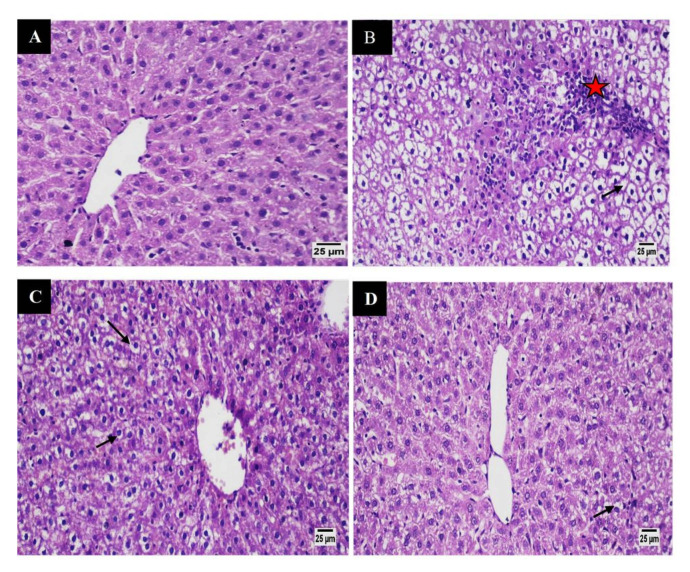
Photomicrographs of H&E-stained sections of liver. (**A**) Control rats showing normal hepatic parenchyma. (**B**) Dia group showing severe vacuolation of the hepatocytes (arrow) with mononuclear inflammatory cells infiltration (red star). (**C**) MTF-treated group showing mild enhancement and mild vacuolation of hepatocytes (arrows). (**D**) MTF-loaded LCS-ALG bead-treated group showing less degree of hepatocellular hypertrophy (arrow).

Histopathological examination of the control group revealed normal hepatic architecture with normal plates of hepatocytes radiating from the central vein (Figure 14A). In contrast, the liver section of Dia rats showed some histopathological changes, such as severe hepatocellular vacuolation, in which cells were swollen with clear cytoplasm plus increased inflammatory cell infiltration (Figure 14B). MTF administration (Group 4) reduced some hepatic damage in which the hepatocytes in the liver section showed mild vacuolation (Figure 14C). The MTF-loaded LCS-ALG beads (Group 5) demonstrated a very low percentage pattern of hepatocellular vacuolation, with several sections examined showing apparently normal hepatic parenchyma (Figure 14D).

Biochemical studies, obtained in the current study, supported the histopathological observations of the liver. According to a previous research [125], the histopathological examination of diabetic rats revealed abnormalities such as swollen hepatocytes with severe cytoplasmic vacuolation, increased inflammatory cell infiltration, and loss of architecture. Brownlee et al. [126] concluded that the hyperglycemia-induced oxidative stress promotes necrosis and apoptosis in all body cells. Our study revealed that treatment with MTF and MTF-loaded LCS-ALG beads, on the other hand, reduced histopathological changes compared to Dia rats, with more enhancement. This could be related to the MTF’s ability to increase antioxidant levels while decreasing the oxidative stress and liver inflammation, as previously reported [122,127].

##### Pancreas

**Figure 15:**
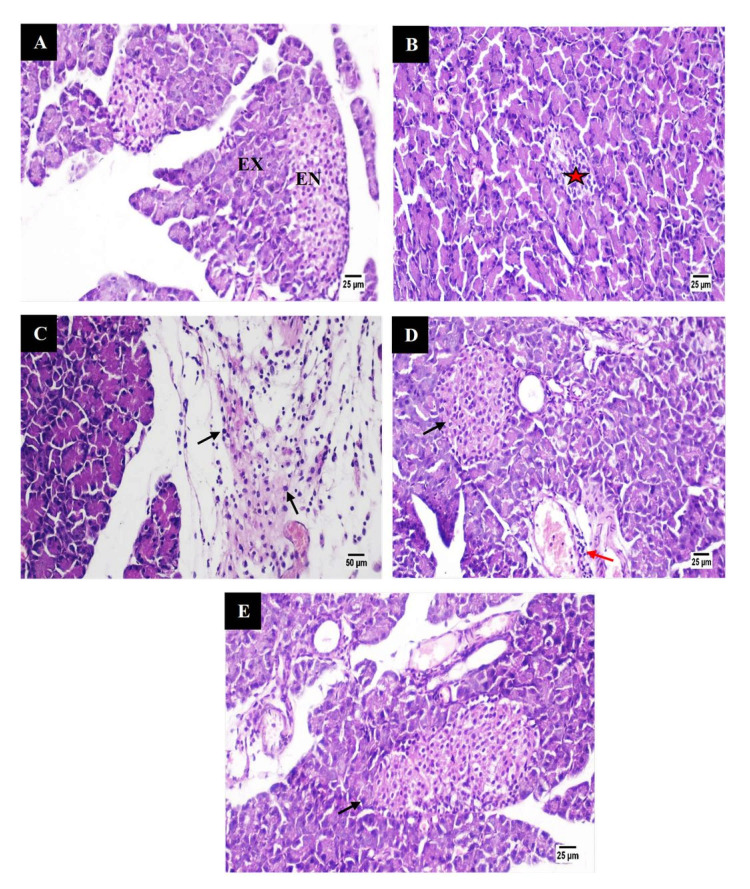
Photomicrographs of H&E-stained sections of pancreas. (**A**) Control rats showing the endocrine pancreas (EN) with histologic features of normal islets. The exocrine pancreatic acini (EX) appear with normal pyramidal lining cells, showing basal basophilia and apical acidophilia. (**B**,**C**) The diabetic group showing small ill-distinct islets containing vacuolated and necrotic cells (red star) and inflammatory cell infiltration (arrows). (**D**) MTF-treated group showing an apparent improvement in area occupied by the islet (black arrow) with mild perivascular mononuclear inflammatory cells infiltration (red arrow). (**E**) MTF-loaded LCS-ALG bead-treated group showing apparently normal islets of Langerhans (arrow).

Microscopic examination of the pancreas from the control group revealed a normal structure of both exocrine units and endocrine components. Islets of Langerhans appeared with normal size and as pale areas between acini (Figure 15A). The Dia group showed small-sized ill-distinct islets of Langerhans, contained few vacuolated and necrotic β-cells. Some of the examined sections exhibited an inflammatory reaction in the peripancreatic tissue (Figure 15B,C). The MTF treatment showed average sized islets with a mild perivascular mononuclear inflammatory cell infiltration (Figure 15D). Concerning the MTF-loaded LCS-ALG bead group, islets of Langerhans were apparently normal with average size and intact cells, and no obvious histological changes were seen in the surrounding pancreatic acini of different sections of all specimens (Figure 15E). Figure S13 in (ESI) shows that the area of islets of Langerhans decreased significantly in the Dia group when compared to the other experimental groups. Both MTF and MTF-loaded LCS-ALG bead-treated groups showed non-significant changes in the area of islets compared to the control group.

Halban et al. [128] concluded that pancreatic β-cell injury was caused by a variety of strategies, such as oxidative damage, increased metabolic stress, increased endoplasmic reticulum injury, and inflammatory pathway activation. According to reported experimental and clinical studies [126,129], hyperglycemia is associated with elevated levels of free radicals, as well as with increased protein expression of inflammatory markers such as nuclear factor kappa B (NF-κB), tumor necrosis factor alfa (TNFα), and interleukin 6 (IL-6) in the pancreas, leading to pancreatic β-cell injury. In accordance with previous studies [129,130], a reduction in the area of islets of the Dia group compared to control animals was observed, indicating a decrease in serum insulin levels and an increase in blood glucose levels in comparison to the control group, consistent with our biochemical findings. However, the MTF treatment showed an amelioration of the histological structure of the pancreas with a mild perivascular mononuclear inflammatory cell infiltration. The beneficial effects of MTF were confirmed through the reduction of oxidative stress, inflammation, and endoplasmic reticulum stress [131]. Concretely, MTF-loaded LCS-ALG beads showed improvement in both the endocrine and exocrine pancreases.

##### Kidneys

**Figure 16:**
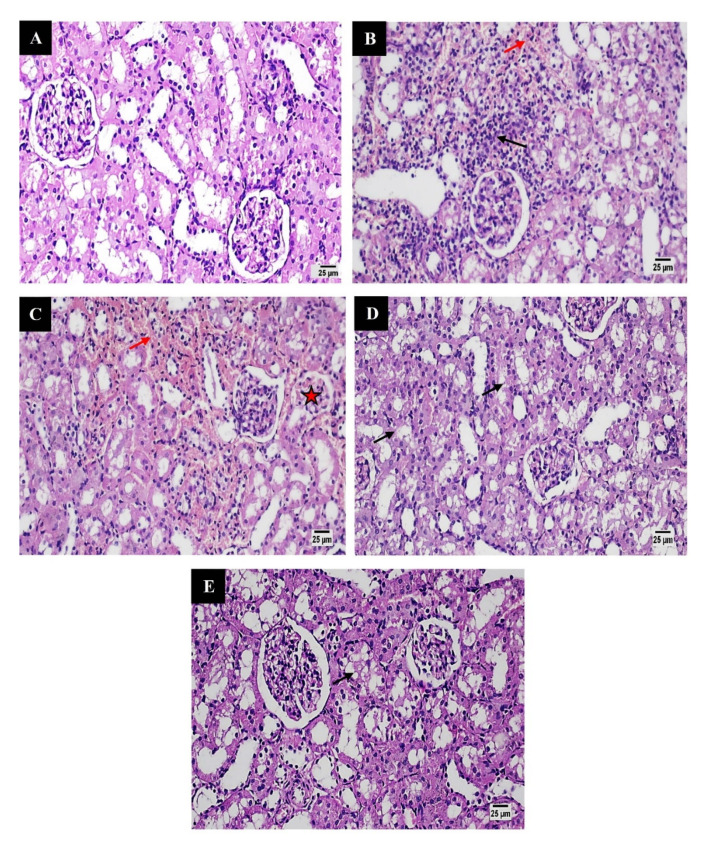
Photomicrographs of H&E-stained sections of kidney. (**A**) Control rat sample showing normal histology of renal cortex. (**B**,**C**) The diabetic rat group sample showing degenerating renal tubules with mononuclear inflammatory cells infiltration (black arrow), interstitial haemorrhage (red arrows), and deformity of renal corpuscle with atrophy of glomerular capillaries (red star). (**D**) MTF-treated group sample showing vacuolation in the renal tubular epithelium (arrows). (**E**) MTF-loaded LCS-ALG bead-treated group sample showing enhancement along with mild vacuolation in the renal tubular epithelium (arrow) with normal renal capsule.

Microscopic examinations of histological sections of kidneys of the control group revealed normal renal glomeruli with normal Bowman’s capsule. In addition, proximal and distal tubules appeared structurally normal (Figure 16A). The Dia group, on the other hand, showed deformed renal corpuscles with glomerular capillary atrophy in some sections, as well as an extensive degeneration in the epithelial lining of renal tubules. There was also evidence of interstitial nephritis, as demonstrated by the presence of mononuclear inflammatory cell infiltration. Additionally, free red blood cells (RBCs) were detected in the cortex between the renal tubules (Figure 16B,C). Sections of the kidney of the MTF-treated group showed degeneration in the renal tubules (Figure 16D). The MTF-loaded LCS-ALG bead-treated rats showed a significant improvement, with normal renal glomeruli and Bowman’s capsule with only mild degeneration in the renal tubule lining (Figure 16E). Diabetic nephropathy represents roughly 30% of people with T1DM and 40% of people with T2DM [10]. In the current study, Dia rats showed kidney histopathological alterations marked by degenerating renal tubules with a mononuclear inflammatory cell infiltration, which was consistent with previous findings [132]. The current study found that the dose of MTF used could, to some extent, alleviate pathological changes in the kidneys of diabetic rats. The molecular mechanism of MTF’s renoprotective impacts is complicated and not fully understood. However, various studies demonstrated that the activation of AMP-activated protein kinase (AMPK) in kidney cells and the consequent enhancement of mitochondrial biogenesis could play a key role [133]. Other evidence suggested that MTF protects renal tubules from injury by restoring the biochemical alterations and regulating the oxidative stress [112]. Another study found that MTF increases the expression of anti-oxidative genes while inhibiting the pro-inflammatory genes in diabetic rat nephropathy [134]. The current study revealed that the MTF-loaded LCS-ALG beads noticeably preserved the kidney’s histology in the treated group, apart from some observed vacuolation in the renal tubular epithelium.

##### Testes

Under light microscopy, abundant seminiferous tubules with large diameters were observed, packed by an increased number of spermatogonia cells and Sertoli cells with numerous spermatids in the control group’s testicular structure (Figure S14A, ESI). Compared to the control group, obvious pathological changes were observed in testicular tissue of Dia group, characterized by small and distorted seminiferous tubules with irregular outlines. Additionally, the number of spermatogenic series cells was decreased with the presence of numerous empty tubules (Figure S14B, ESI). The Dia group, treated with MTF, showed mildly degenerating seminiferous tubules manifested by the decrease in the number of the spermatogenic series. Therefore, the MTF treatment partially restored testicular tissue (Figure S14C, ESI). However, MTF-loaded LCS-ALG bead treatment protected the testicular tissue from diabetes-induced damage, so it appeared normal in almost all the examined sections apart from some degeneration in the spermatogonia cells (Figure S14D, ESI).

In the current study, alterations in testicular morphology, including greatly irregularly placed small-sized seminiferous tubules with large interstitial space along with impaired and not completed spermatogenesis, were observed in the Dia rats. These findings were consistent with Guneli et al. [135], who reported that hyperglycemia reduces the spermatogenesis as well as the diameter of the seminiferous tubules. Our findings showed that the Dia group exhibits lower levels of serum testosterone, which is required for spermatogenesis and the development of the secondary sexual characteristics. Reddy et al. [113] concluded that a potential method of spermatogenesis reduction in diabetic animals was associated with reduced steroidogenesis as well as elevated oxidative stress, leading to testicular dysfunction in the experimental rats.

Consistent with current results, Gholizadeh et al. [136] reported that MTF improved many aspects of diabetes-induced testicular dysfunction such as the testis weight and volume and sperm count and motility. In line with that, Faure et al. [137] reported that MTF has critical effects on steroidogenesis and spermatogenesis in testicular tissue, particularly through the activation of the AMPK pathway, which induces lactate production without affecting lactate dehydrogenase concentration in cultured Sertoli cells, though the exact molecular mechanism and the action of MTF remains unclear. Our findings showed that MTF-loaded LCS-ALG beads are effective in maintaining rat testicular tissue to a significant degree. In short, histopathological studies indicated that MTF-loaded LCS-ALG beads extremely reduced the degenerative changes of the liver, pancreas, kidney, and testes.

## 4. Conclusions

A versatile plant-pollen-derived protective dual microencapsulation platform for the first-line antidiabetic drug metformin (MTF) was developed for enhanced bioavailability. MTF was encapsulated into the LCS sporopollenin and raw DPP pollens using vacuum and natural hydration-induced swelling techniques, respectively. The dual encapsulation platform for MTF was optimized via re-encapsulation of the MTF-loaded LCS into ALG beads. The optimized MTF-loaded LCS-ALG beads exhibited the slowest and most sustained in vitro MTF release profile, where 14% of MTF was slowly released in SGF within 2 h, followed by a faster release in SIF releasing 54% at 9.5 h, which could be beneficial for advanced patient compliance and reduced dosing frequency. The release kinetics data for the optimized beads revealed that Fickian diffusion mechanism was ascribed in SGF, whereas in SIF, the release followed zero-order kinetics, indicating a controlled release with the super case II transport mechanism. The optimized bead formula showed bioavailability of 122% relative to pure MTF. Relative to the STZ-induced diabetic rat model, a significant hypoglycemic effect was obtained for the optimized MTF-loaded LCS-ALG beads, where the BGLs were maintained after 14 days, reaching 71% reduction at Day 29. Our results demonstrated that these optimized beads could also be used as a novel strategy for controlling lipid peroxidation and maintaining lipid, lipoprotein, and renal parameters. In addition, antioxidant and anti-inflammatory properties of MTF-loaded LCS-ALG beads improved the histological changes in diabetic rats’ liver, pancreas, kidney, and testes. This study offers a promising untapped potential therapeutic approach to MTF by combining the benefits of plant-pollen-derived biomaterials and ALG polymer for the management of T2DM complications, paving the way for potential intriguing applications in sustained drug delivery.

## Figures and Tables

**Table 1:** Loading capacity (%LC), encapsulation efficiency (%EE), and production yield for MTF-loaded LCS, MTF-loaded DPP, and MTF-loaded LCS-ALG beads. Data are presented as (mean ± SE, *n* = 3).

	MTF-Loaded LCS	MTF-Loaded DPP	MTF-Loaded LCS-ALG Beads
%LC	14.91 ± 0.65	15.17 ± 0.72	10.00 ± 0.80
%EE	29.83 ± 0.83	30.34 ± 1.02	24.45 ± 1.95
%Yield	68.50 ± 5.21	66.10 ± 4.68	74.30 ± 3.24

**Table 2:** PK parameters of MTF in plasma following oral administration of single dose of pure MTF and the optimized MTF-loaded LCS-ALG beads equivalent to 25 mg/kg bw.

Pharmacokinetic Parameter	Pure MTF	MTF-Loaded LCS-ALG Beads
AUC_0–10_ (ng.h/mL)	9453.85	10982.46
AUC_0__–__∞_ (ng.h/mL)	9718.15	11808.68
C_max_ (ng/mL)	1618.00	2128.50
t_1/2_ (h)	1.425	1.571
K_el_ (h^−1^)	0.49	0.44
T_max_ (h)	2.00	6.00
MRT_0–10_ (h)	3.97	6.13

AUC_0–t_, area under the plasma concentration–time curve from zero to 10 h postdose; AUC_0–∞_, area under the plasma concentration–time curve from zero to infinity; C_max_, maximum plasma concentration; t_1/2_, terminal phase half-life; T_max_, time to reach C_max_; k_el_, elimination rate constant; MRT_0–10_, mean residence time.

## Data Availability

Available upon request from the authors.

## Supplementary Materials

The following are available online at https://www.mdpi.com/article/10.3390/pharmaceutics13071048/s1, Supplementary materials include Figures S1–S14, Table S1 and Movies 1,2. Figure S1: Size distribution of inflated raw DPP pollen in water D(0.5) = 19.346 mm. The size distribution was measured using (Zetasizer Nano ZL, Malvern, UK), Figure S2: (A) Optical image of raw DPPs dispersed in 10% *w*/*v* CTAB aqueous solution and then dried (B) SEM image of raw DPPs after treated with 10% *w*/*v* CaCl2 and then dried. (C) Optical image showing the interface between dry and hydrated raw DPPs, where they were elliptically and spherically shaped, respectively, Figure S3: SEM images showing the ornamentations and surface morphology of different parts of raw DPP pollen surfaces. Some pollens show shapes resembling a folded pita-bread with a thickness of around 1 mm. The perforated decorations showed grooves with different sizes, Figure S4: Energy-dispersive X-ray spectroscopy (EDX) spectra imaging for different parts of inflated raw DPP spores. (A) inner grey mass, (B) the outer shell (exine), (C) dark spots inside the protoplast (enclosed within the intine of the pollen and (D) inner (intine) layer, Figure S5: Digital and optical images of the MTF-loaded LCS-ALG beads during, after preparation and after drying in air for 24 h, Figure S6: SEM images of MTF-loaded LCS-ALG beads. Few manually broken (by spatula) beads are shown with arrows. Some beads have small tails formed while extruding from the 23G needle, Figure S7: SEM images of LCS microcapsules left from MTF-loaded LCS-ALG beads after the 4th sonication recovery cycle during the MTF loading capacity (LC) test. Some surface damages of the ornamentation features can be seen (arrows), Figure S8: FTIR spectrum for pure MTF, LCS, MTF-loaded LCS, raw DPP, MTF-loaded DPP, pure alginate beads ALG, MTF-loaded LCS-ALG beads and pure sodium alginate (SA), Figure S9: (A) TGA thermograms for pure MTF, LCS, MTF-loaded LCS, raw DPP, MTF-loaded DPP, pure alginate beads ALG and MTF-loaded LCS-ALG beads. (B) Derivative TG for formulations involved LCS microcapsules. (C) Derivative TG for formulations involved DPP microcapsules, Figure S10: Rats weight over 29 days, data are presented as (mean ± SE, *n* = 6), significance at *p* < 0.05. ^a^: significantly different when compared to control, ^b^: significantly different when compared to LCS + ALG, ^c^: significantly different when compared to Dia, ^d^: significantly different when compared to compared to Dia + MTF and ^e^: significantly different when compared to MTF-Loaded LCS-ALG beads group, Figure S11: Lipid profile at the end of the treatment period (29 days). (A) Serum levels of total cholesterol. (B) triglycerides. (C) HDL-C. (D) LDL-C. Data are presented as (mean ± SE, *n* = 6), significance at *p* < 0.05. ^a^: significantly different when compared to control, ^b^: significantly different when compared to LCS + ALG, ^c^: significantly different when compared to Dia, ^d^: significantly different when compared to compared to Dia + MTF and ^e^: significantly different when compared to MTF-loaded LCS-ALG beads group, Figure S12: Liver and kidney parameters at the end of the treatment period (29 days). (A) Serum levels of ALT. (B) AST. (C) Creatinine. (D) Urea. (E) Uric acid. Data are presented as (mean ± SE, *n* = 6), significance at *p* < 0.05. ^a^: significantly different when compared to control, ^b^: significantly different when compared to LCS + ALG, ^c^: significantly different when compared to Dia, ^d^: significantly different when compared to compared to Dia + MTF and ^e^: significantly different when compared to MTF-loaded LCS-ALG beads group, Figure S13: Area of islets of Langerhans in different groups. Data are presented as (mean ± SE, *n* = 6), significance at *p* < 0.05. ^a^: significantly different when compared to control, ^b^: significantly different when compared to Dia, ^c^: significantly different when compared to compared to Dia + MTF and ^d^: significantly different when compared to MTF-loaded LCS-ALG beads group, Figure S14: Photomicrographs of H&E-stained sections of testes. (A) Testicular sections of a control rat showing normal seminiferous tubules with normal spermatogenesis (arrows). (B) Testicular sections of a diabetic rat showing atrophic, irregular seminiferous tubules with smaller diameters (arrows). (C) Testicular section of a MTF group showing seminiferous tubular structures with degeneration of spermatogenic cells in some seminiferous tubules (arrows). (D) Testicular sections of MTF-loaded LCS-ALG beads group showing apparently normal seminiferous tubules with normal diameters and spermatogenic cells in almost all the seminiferous tubules, Table S1: Release kinetics models of MTF from different formulations at different pHs, Movie Files: Video 1: Date Palm pollens after loading with the MTF, Video 2: Swelling of the hydrated Date palm pollens, which attain their original oval shape again after drying.
