# Supplementary material for: Sustained In Vitro and In Vivo Delivery of Metformin from Plant Pollen-Derived Composite Microcapsules
Source: Pharmaceutics. 2021 Jul 9;13(7):1048. doi: 10.3390/pharmaceutics13071048 (PMC8309045; doi:10.3390/pharmaceutics13071048)
Supplement: Supplementary file 1 [file pharmaceutics-13-01048-s001.zip › pharmaceutics-1250653-supplementary.pdf]

pharmaceutics

Supplementary Material: Sustained In Vitro and In Vivo Delivery of Metformin from Plant Pollen-Derived Composite Microcapsules

Noha M. Meligi, Amro K.F. Dyab and Vesselin N. Paunov

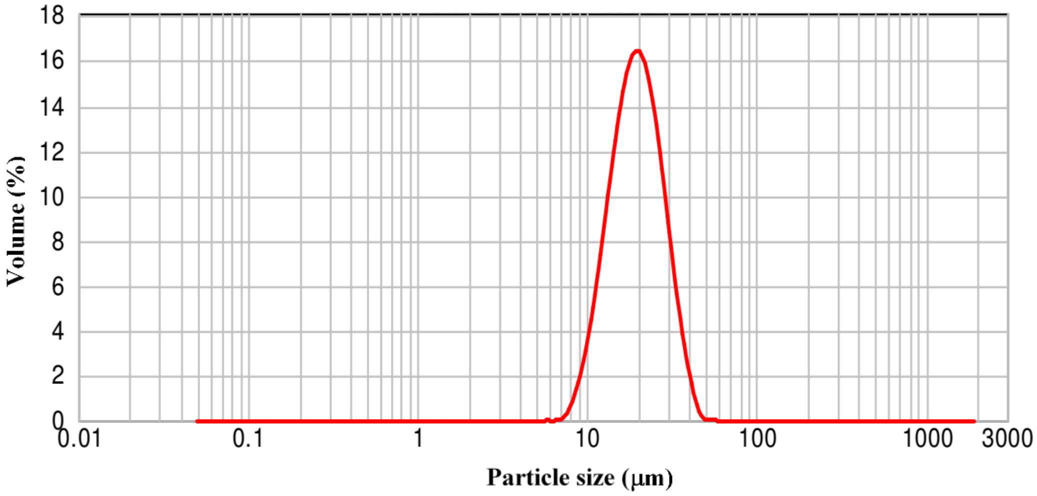

Figure S1. Size distribution of inflated raw DPP pollen in water D(0.5) = 19.346 mm. The size distribution was measured using (Zetasizer Nano ZL, Malvern, UK).

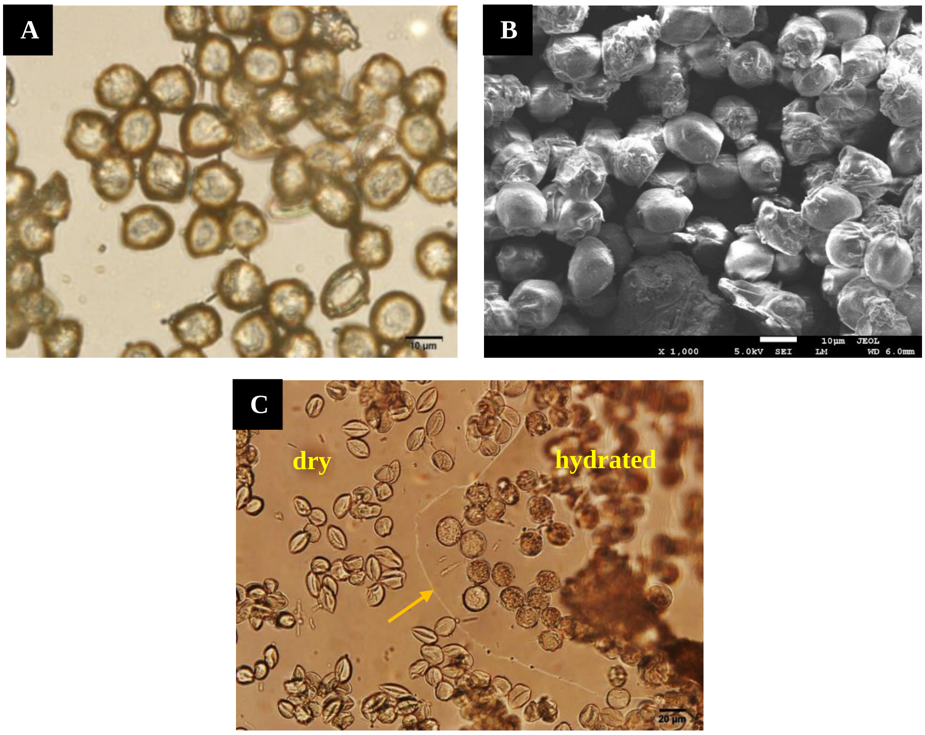

Figure S2. (A) Optical image of raw DPPs dispersed in 10% w/v CTAB aqueous solution and then dried (B) SEM image of raw DPPs after treated with 10% w/v $CaCl_2$ and then dried. (C) Optical image showing the interface between dry and hydrated raw DPPs, where they were elliptically and spherically shaped, respectively.

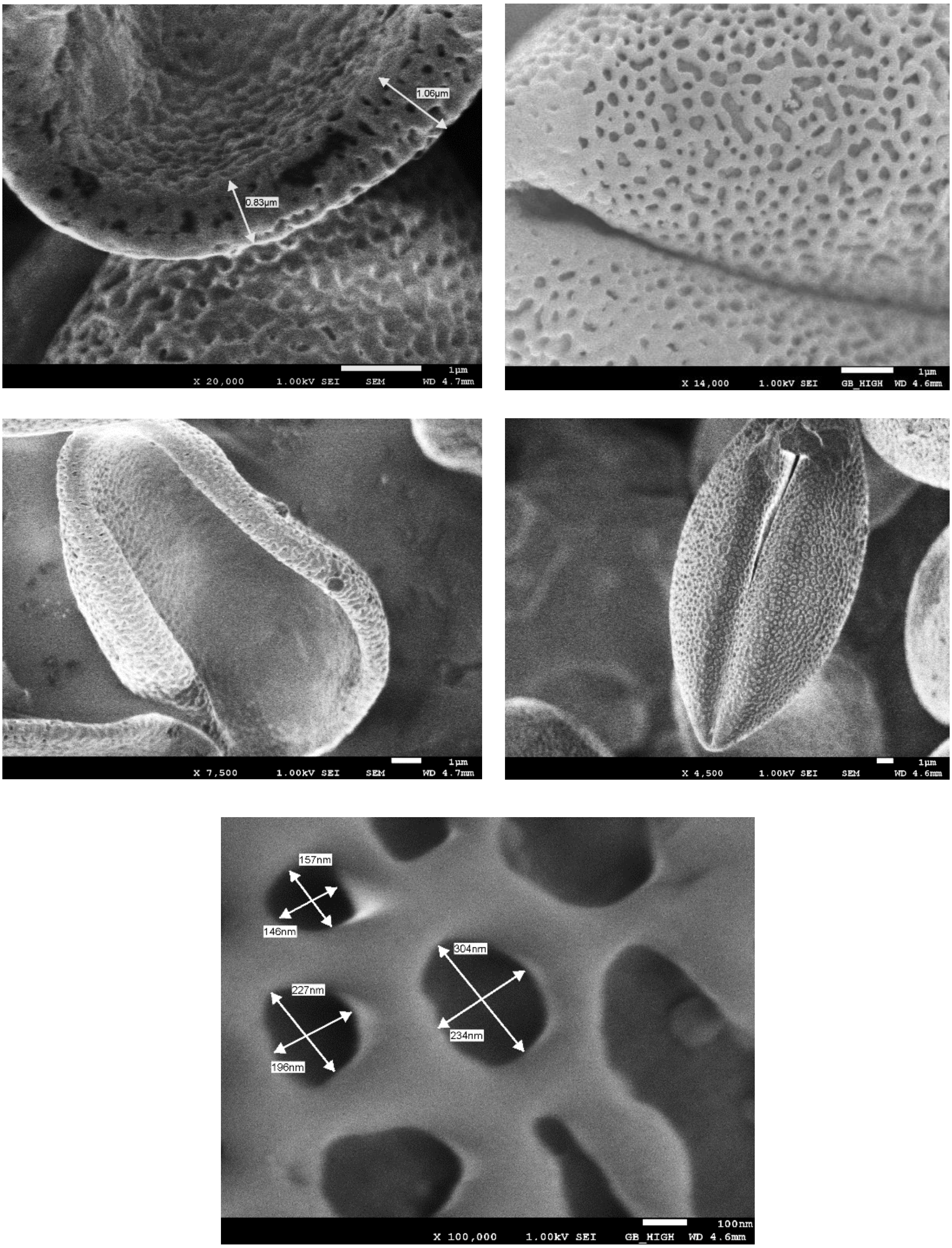

Figure S3. SEM images showing the ornamentations and surface morphology of different parts of raw DPP pollen surfaces. Some pollens show shapes resembling a folded pita-bread with a thickness of around 1 mm. The perforated decorations showed grooves with different sizes.

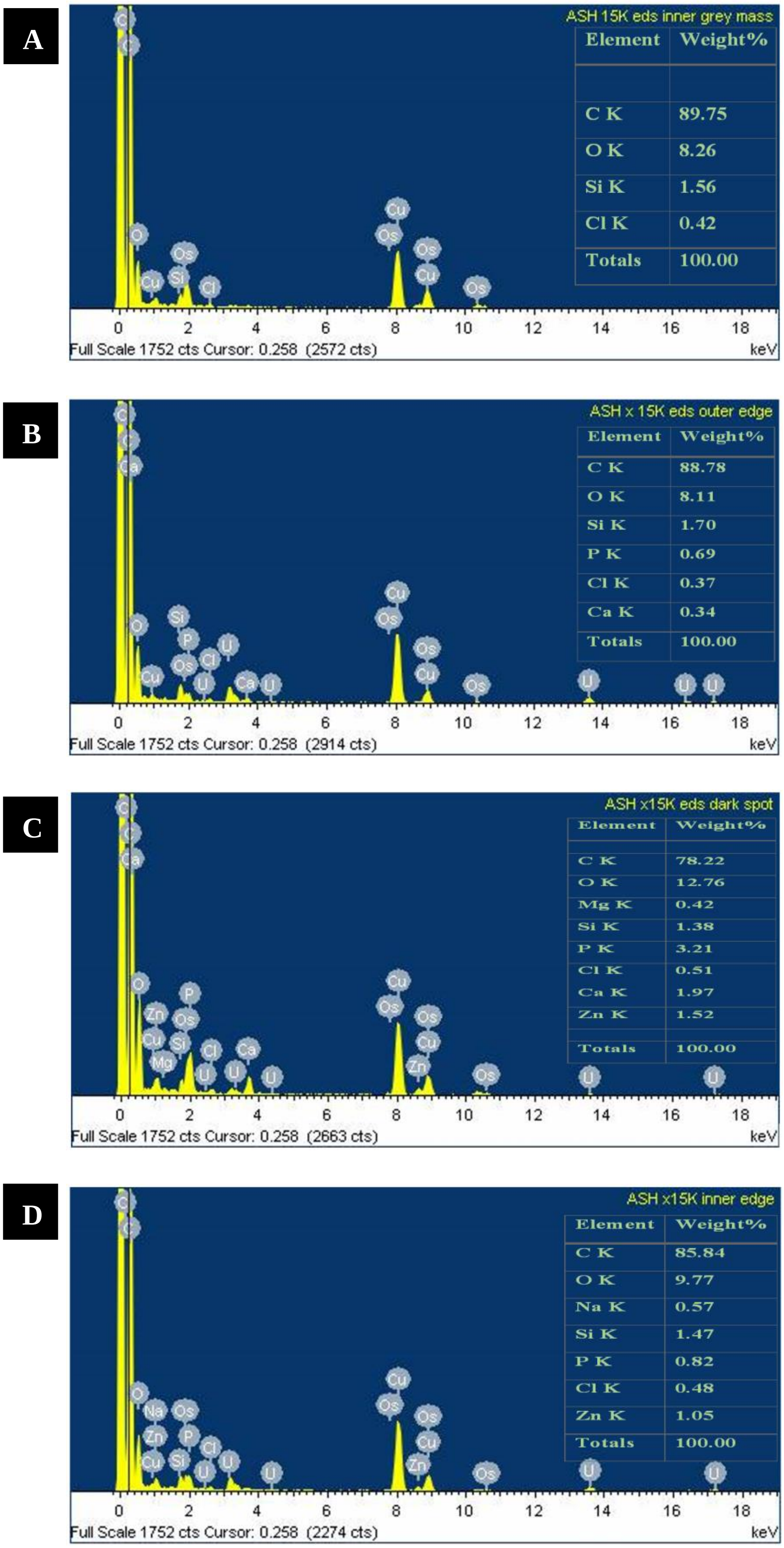

Figure S4. Energy-dispersive X-ray spectroscopy (EDX) spectra imaging for different parts of inflated raw DPP spores. (A) inner grey mass, (B) the outer shell (exine), (C) dark spots inside the protoplast (enclosed within the intine of the pollen and (D) inner (intine) layer.

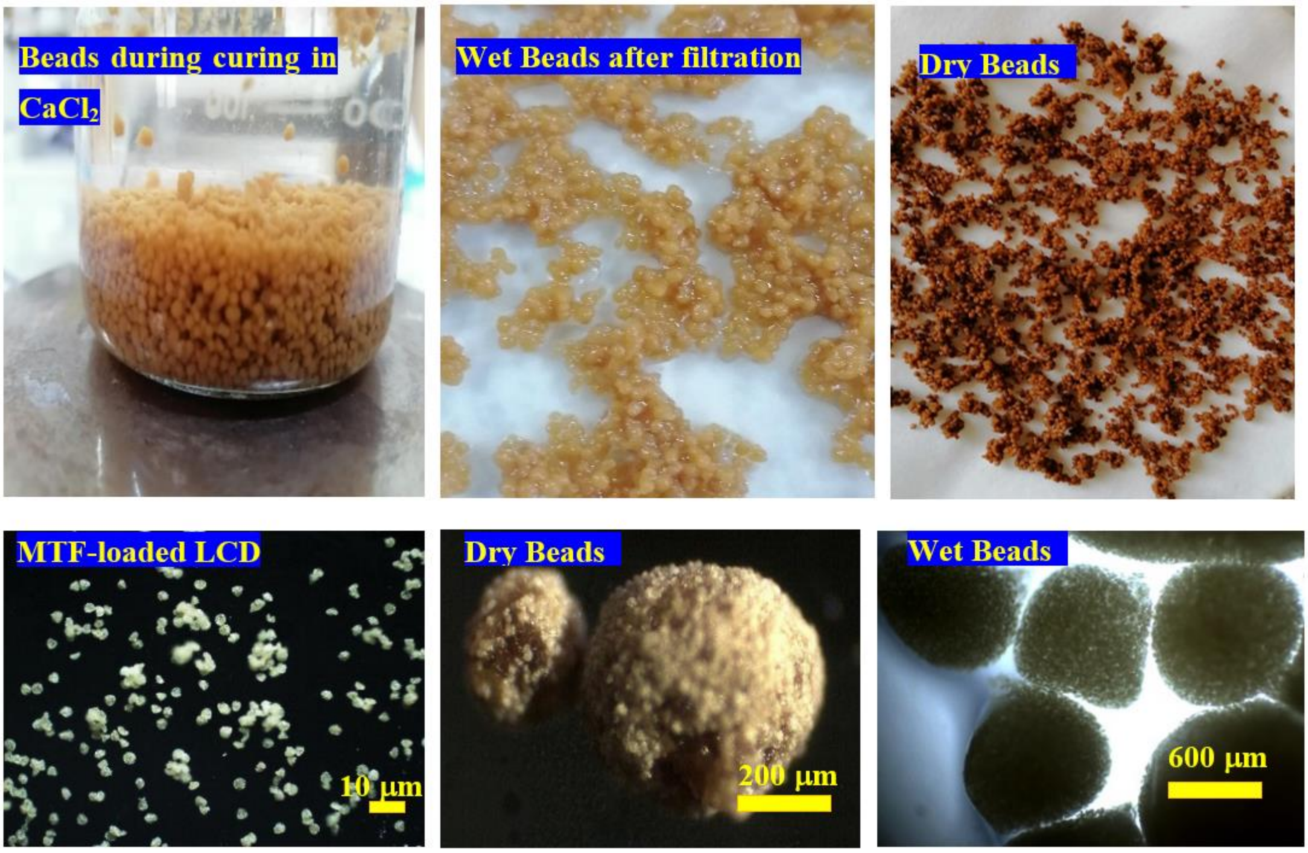

Figure S5. Digital and optical images of the MTF-loaded LCS-ALG beads during, after preparation and after drying in air for 24 h.

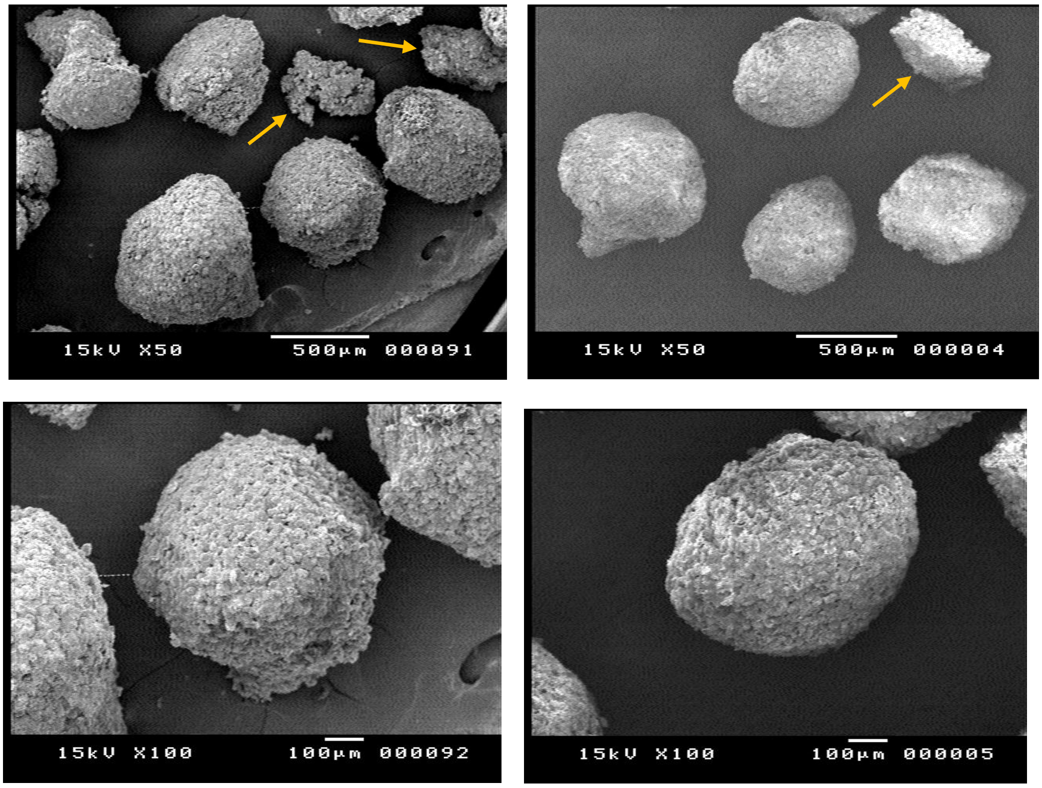

Figure S6. SEM images of MTF-loaded LCS-ALG beads. Few manually broken (by spatula) beads are shown with arrows. Some beads have small tails formed while extruding from the 23G needle.

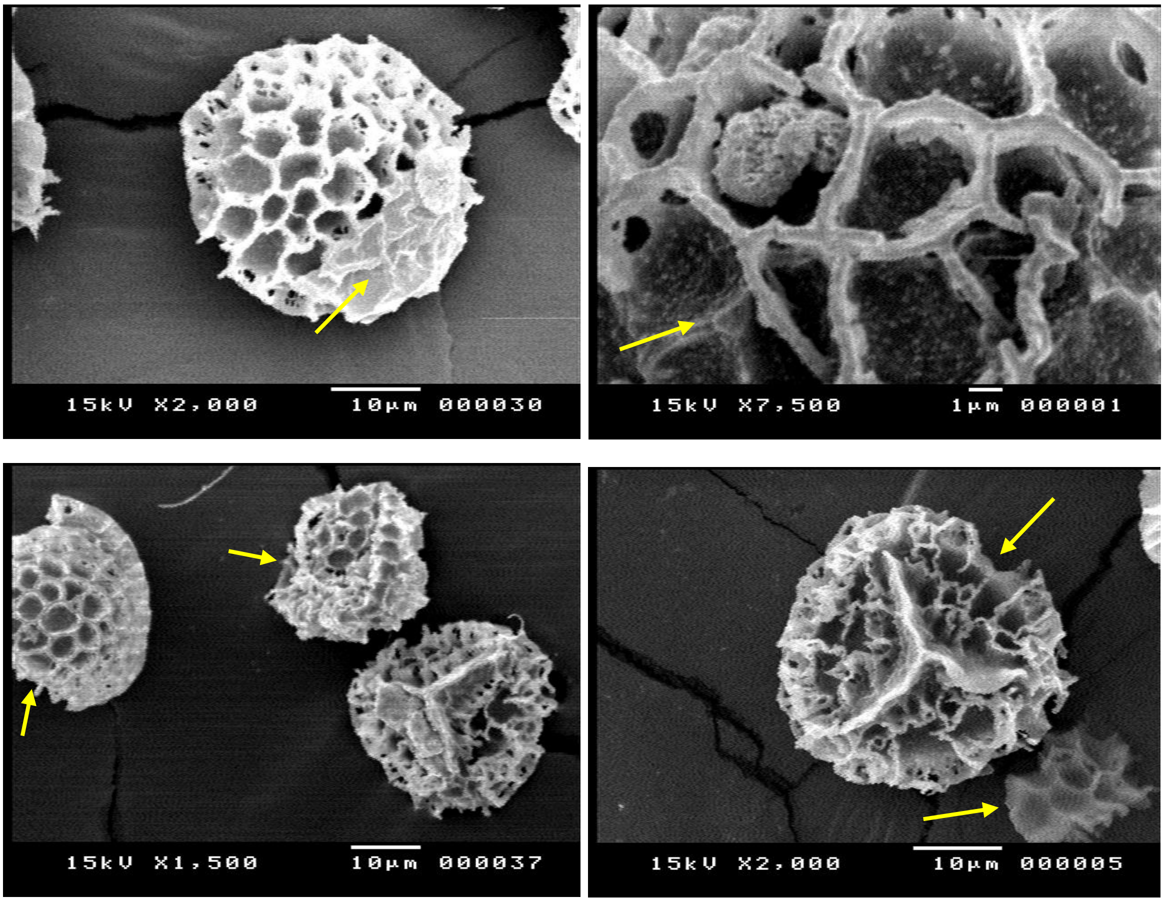

Figure S7. SEM images of LCS microcapsules left from MTF-loaded LCS-ALG beads after the 4th sonication recovery cycle during the MTF loading capacity (LC) test. Some surface damages of the ornamentation features can be seen (arrows).

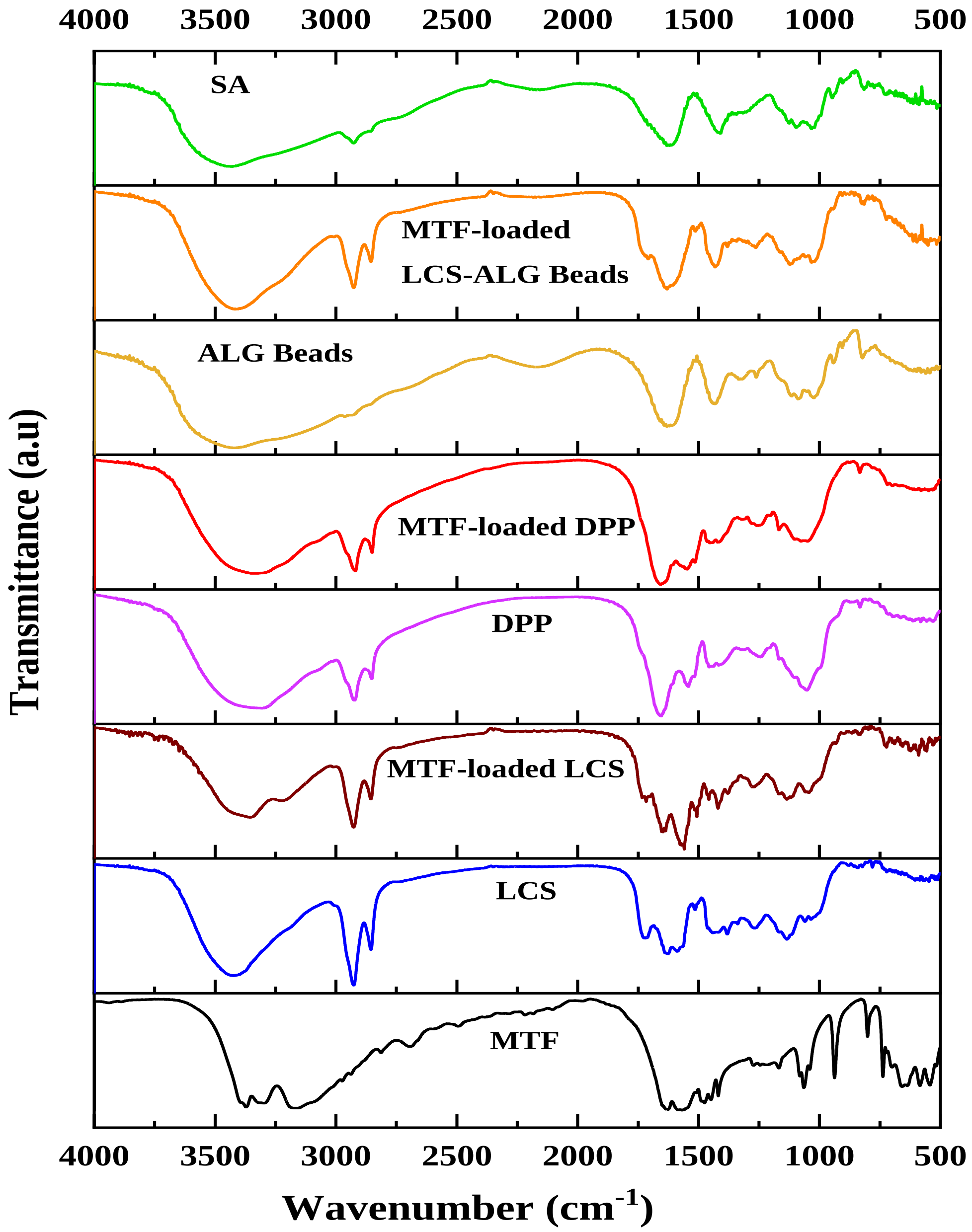

Figure S8. FTIR spectrum for pure MTF, LCS, MTF-loaded LCS, raw DPP, MTF-loaded DPP, pure alginate beads ALG, MTF-loaded LCS-ALG beads and pure sodium alginate (SA).

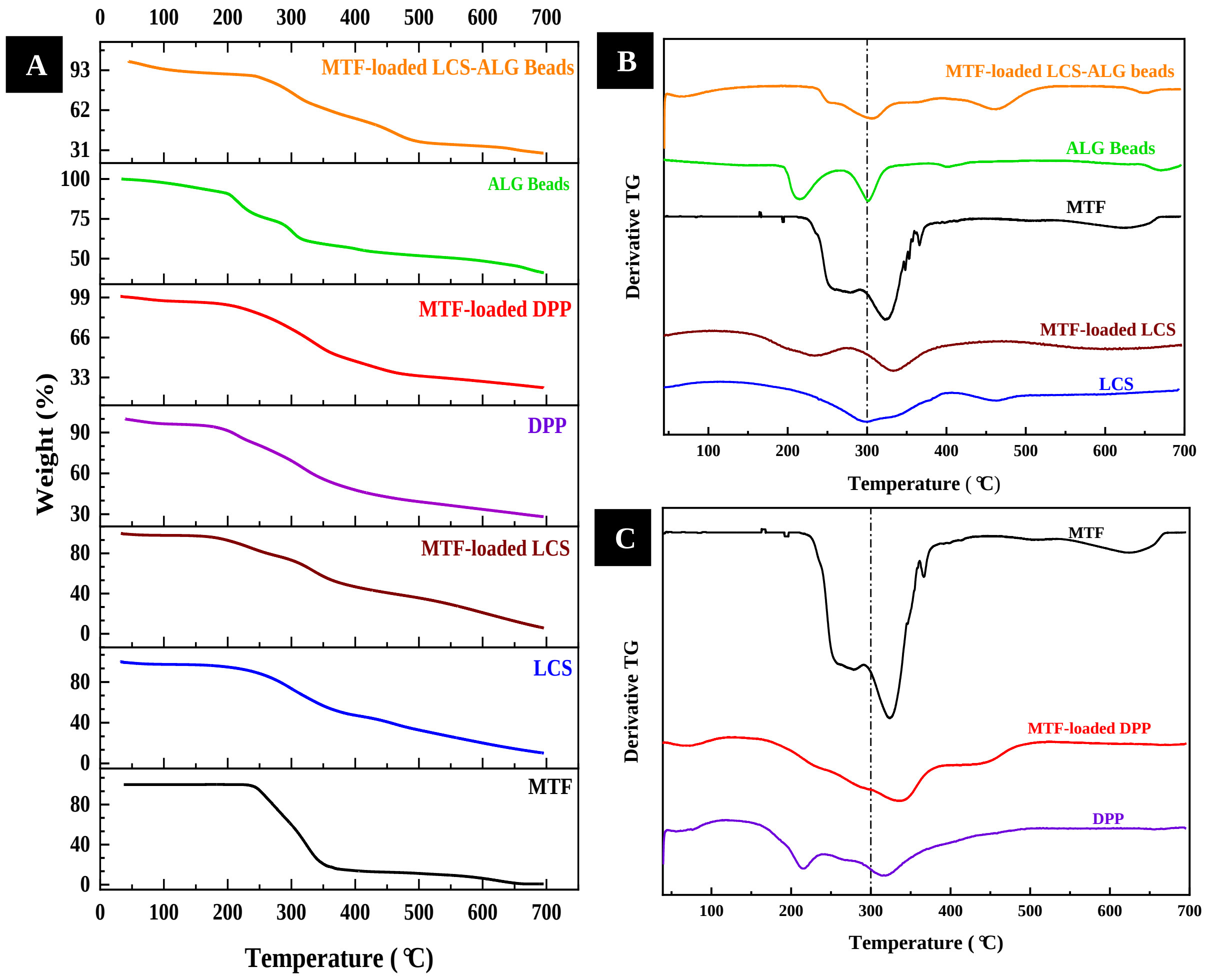

Figure S9. (A) TGA thermograms for pure MTF, LCS, MTF-loaded LCS, raw DPP, MTF-loaded DPP, pure alginate beads ALG and MTF-loaded LCS-ALG beads. (B) Derivative TG for formulations involved LCS microcapsules. (C) Derivative TG for formulations involved DPP microcapsules.

Table S1. Release kinetics models of MTF from different formulations at different pHs.

Formulation/media	Zero-order		First-order		Higuchi		Korsmeyer-Peppas		
	R^2	k_0	R^2	k_1	R^2	K_H	R^2	k_{kp}	n
MTF-loaded LCS (SGF)	0.8615	7.222	0.875	0.089	0.9377	14.50	0.9571	20.61	0.369
MTF-loaded LCS (SIF)	0.9693	7.311	0.979	0.198	0.9829	35.38	0.9664	15.51	0.758
MTF-loaded DPP (SGF)	0.8587	5.139	0.868	0.060	0.9334	10.32	0.9539	15.71	0.342
MTF-loaded DPP (SIF)	0.970	7.26	0.9835	0.163	0.983	35.12	0.9603	11.12	0.870
MTF-loaded LCS-ALG (SGF)	0.8698	4.962	0.8745	0.055	0.9381	9.92	0.9647	10.690	0.495
MTF-loaded LCS-ALG (SIF)	0.9886	5.226	0.9769	0.082	0.9813	25.02	0.9886	6.699	0.910
**Pure MTF (SGF)**	0.8723	4.465	0.9814	0.153	0.982	17.34	0.9606	53.55	0.178
**Pure MTF (SIF)**	0.8172	5.715	0.9942	0.332	0.9856	20.06	0.9487	59.31	0.110

R^2: correlation coefficient, K: rat constant, n: release exponent.

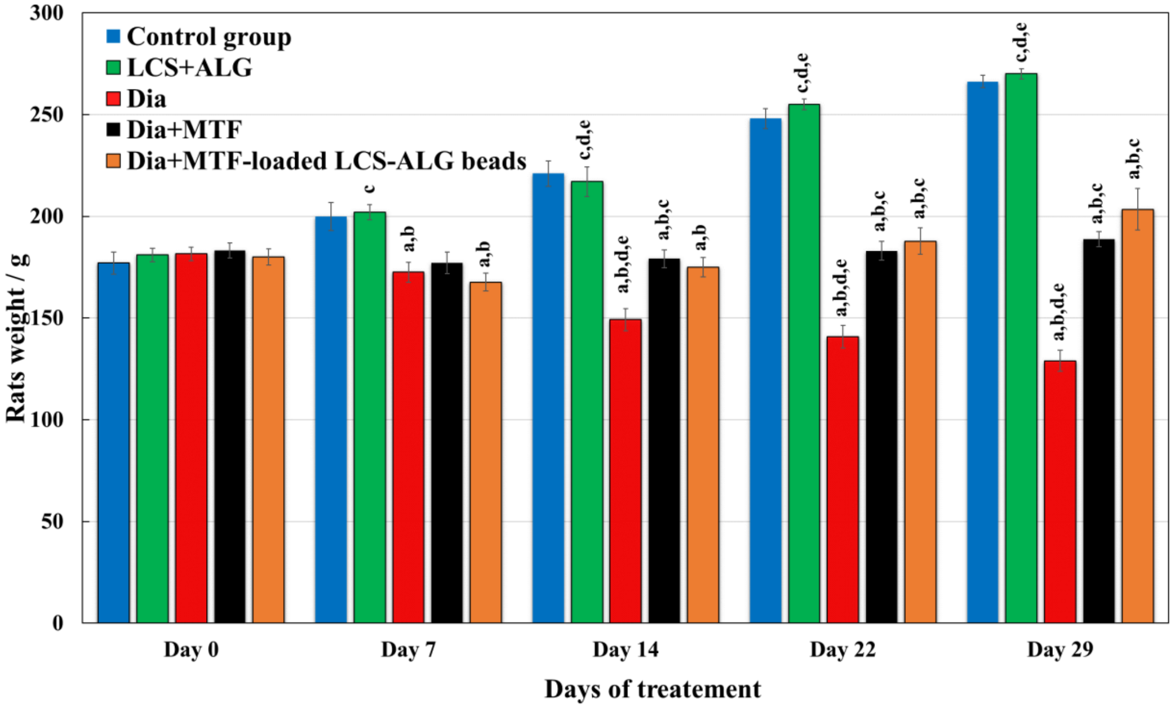

Figure S10. Rats weight over 29 days, data are presented as (mean ± SE, n = 6), significance at P < 0.05. [a]: significantly different when compared to control, [b]: significantly different when compared to LCS+ALG, [c]: significantly different when compared to Dia, [d]: significantly different when compared to compared to Dia+MTF and [e]: significantly different when compared to MTF-Loaded LCS-ALG beads group.

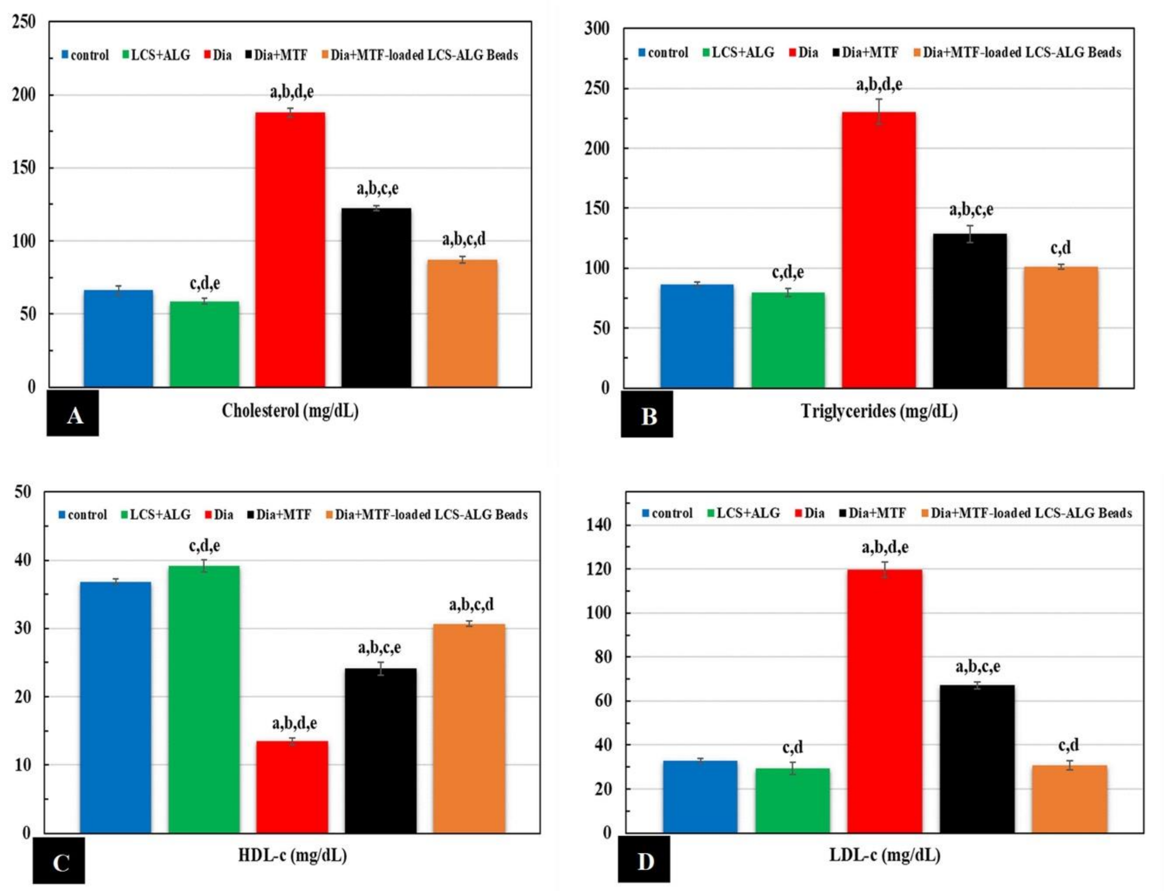

Figure S11. Lipid profile at the end of the treatment period (29 days). (A) Serum levels of total cholesterol. (B) triglycerides. (C) HDL-C. (D) LDL-C. Data are presented as (mean ± SE, n = 6), significance at $P < 0.05$. [a]: significantly different when compared to control, [b]: significantly different when compared to LCS+ALG, [c]: significantly different when compared to Dia, [d]: significantly different when compared to compared to Dia+MTF and [e]: significantly different when compared to MTF-loaded LCS-ALG beads group.

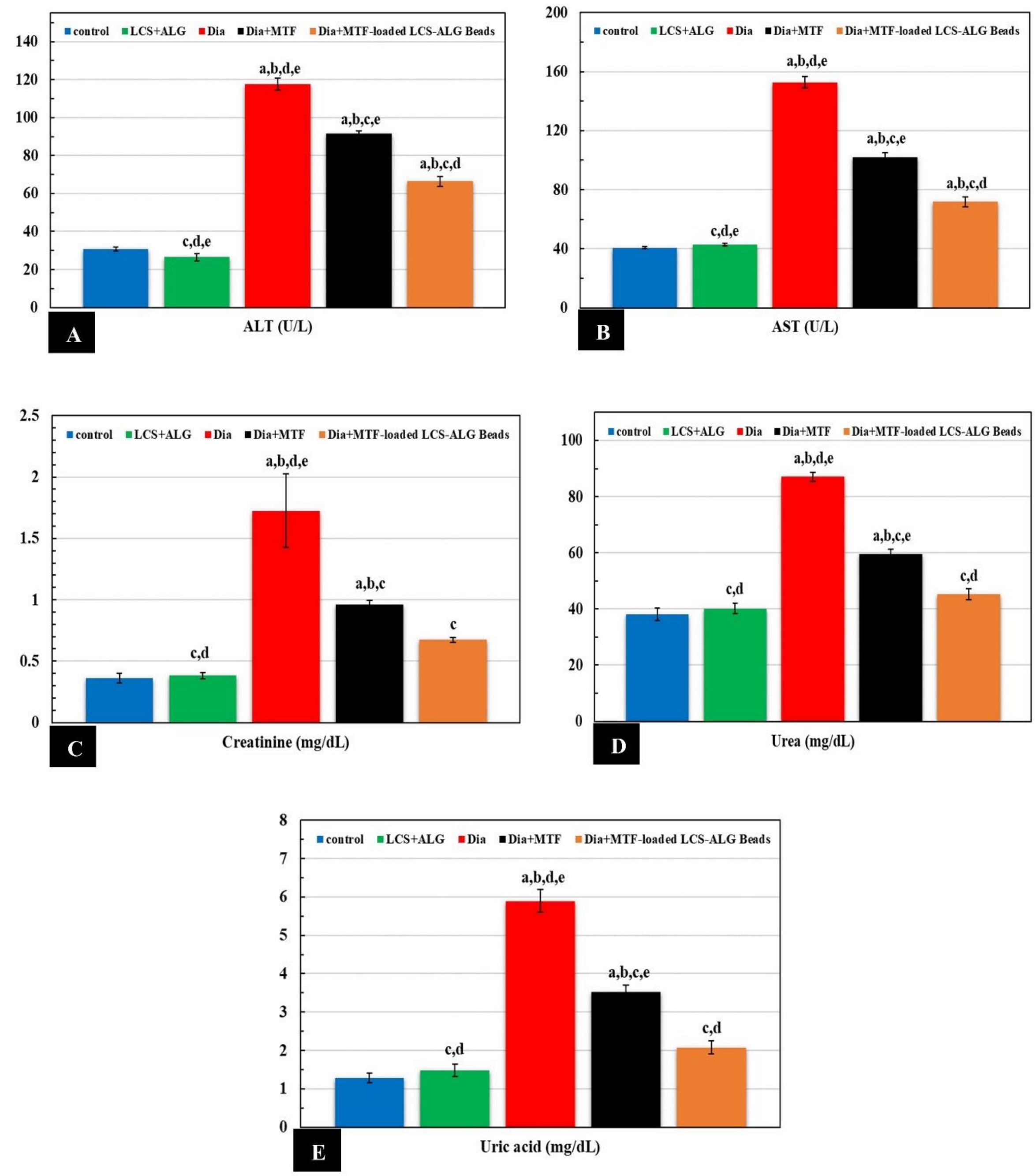

Figure S12. Liver and kidney parameters at the end of the treatment period (29 days). (A) Serum levels of ALT. (B) AST. (C) Creatinine. (D) Urea. (E) Uric acid. Data are presented as (mean ± SE, n = 6), significance at $P < 0.05$. [a]: significantly different when compared to control, [b]: significantly different when compared to LCS+ALG, [c]: significantly different when compared to Dia, [d]: significantly different when compared to compared to Dia+MTF and [e]: significantly different when compared to MTF-loaded LCS-ALG beads group.

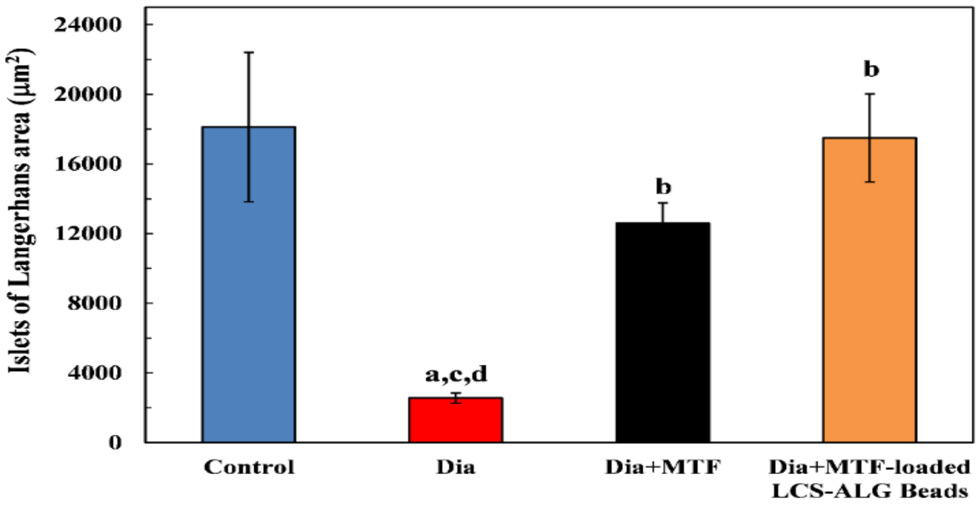

Figure S13. Area of islets of Langerhans in different groups. Data are presented as (mean ± SE, *n* = 6), significance at P < 0.05. [a]: significantly different when compared to control, [b]: significantly different when compared to Dia, [c]: significantly different when compared to compared to Dia+MTF and [d]: significantly different when compared to MTF-loaded LCS-ALG beads group.

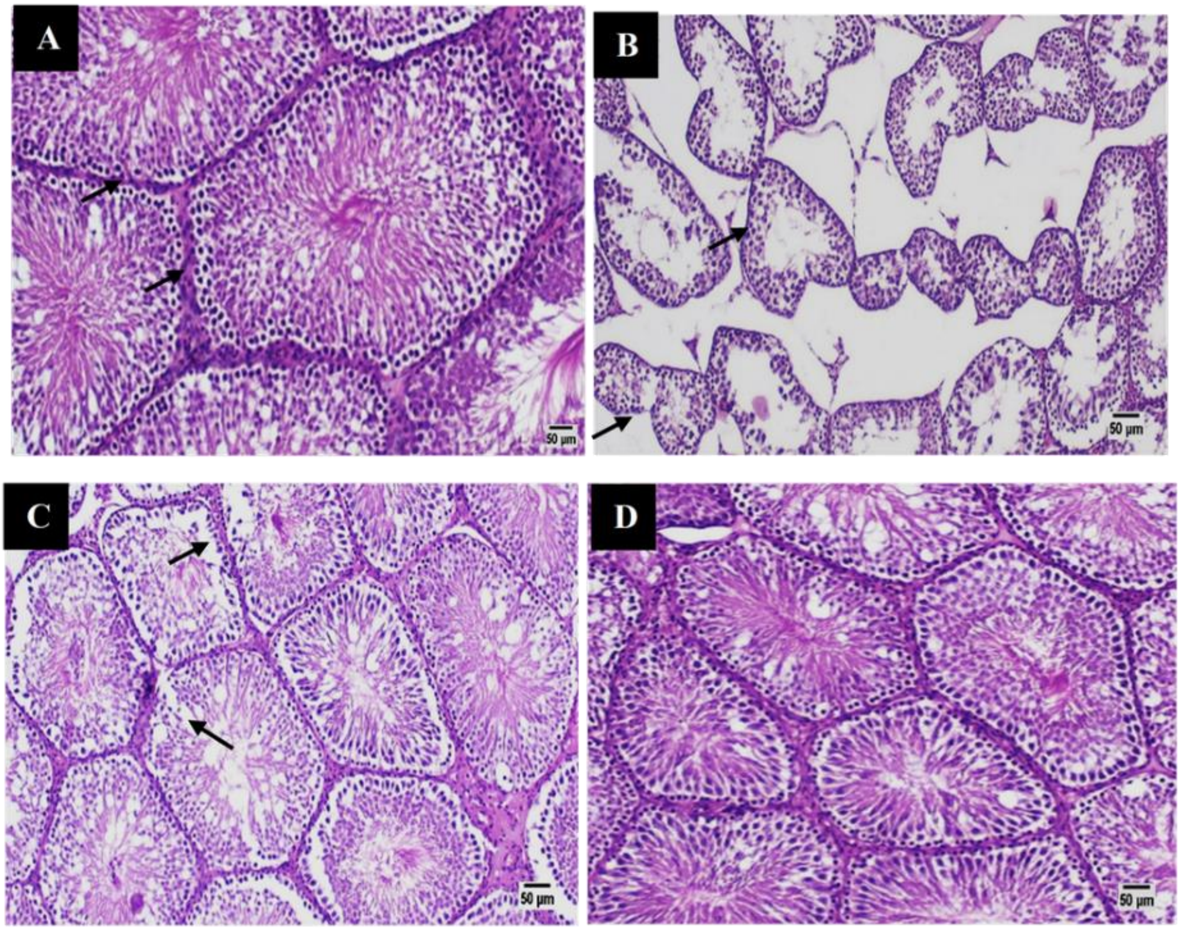

Figure S14. Photomicrographs of H&E-stained sections of testes. (A) Testicular sections of a control rat showing normal seminiferous tubules with normal spermatogenesis (arrows). (B) Testicular sections of a diabetic rat showing atrophic, irregular seminiferous tubules with smaller diameters (arrows). (C) Testicular section of a MTF group showing seminiferous tubular structures with degeneration of spermatogenic cells in some seminiferous tubules (arrows). (D) Testicular sections of MTF-loaded LCS-ALG beads group showing apparently normal seminiferous tubules with normal diameters and spermatogenic cells in almost all the seminiferous tubules.

Movie Files.

Video 1: Date Palm pollens after loading with the MTF.

Video 2: Swelling of the hydrated Date palm pollens, which attain their original oval shape again after drying.
